# Ad26.COV2.S prevents upregulation of SARS-CoV-2 induced pathways of inflammation and thrombosis in hamsters and rhesus macaques

**DOI:** 10.1371/journal.ppat.1009990

**Published:** 2022-04-08

**Authors:** Malika Aid, Samuel J. Vidal, Cesar Piedra-Mora, Sarah Ducat, Chi N. Chan, Stephen Bondoc, Alessandro Colarusso, Carly E. Starke, Michael Nekorchuk, Kathleen Busman-Sahay, Jacob D. Estes, Amanda J. Martinot, Dan H. Barouch

**Affiliations:** 1 Center for Virology and Vaccine Research, Beth Israel Deaconess Medical Center, Boston, Massachusetts, United States of America; 2 Department of Comparative Pathobiology, Section of Pathology, Tufts University Cummings School of Veterinary Medicine, North Grafton, Massachusetts, United States of America; 3 Vaccine & Gene Therapy Institute, Beaverton, Oregon, United States of America; 4 Department of Biochemistry and Molecular Medicine, University of Montreal, Montreal, Canada; 5 Oregon National Primate Research Center, Oregon Health & Sciences University, Beaverton, Oregon, United States of America; 6 Ragon Institute of MGH, MIT, and Harvard, Cambridge, Massachusetts, United States of America; University of Texas Medical Branch / Galveston National Laboratory, UNITED STATES

## Abstract

Syrian golden hamsters exhibit features of severe disease after SARS-CoV-2 WA1/2020 challenge and are therefore useful models of COVID-19 pathogenesis and prevention with vaccines. Recent studies have shown that SARS-CoV-2 infection stimulates type I interferon, myeloid, and inflammatory signatures similar to human disease and that weight loss can be prevented with vaccines. However, the impact of vaccination on transcriptional programs associated with COVID-19 pathogenesis and protective adaptive immune responses is unknown. Here we show that SARS-CoV-2 WA1/2020 challenge in hamsters stimulates myeloid and inflammatory programs as well as signatures of complement and thrombosis associated with human COVID-19. Notably, immunization with Ad26.COV2.S, an adenovirus serotype 26 vector (Ad26)-based vaccine expressing a stabilized SARS-CoV-2 spike protein, prevents the upregulation of these pathways, such that the mRNA expression profiles of vaccinated hamsters are comparable to uninfected animals. Using proteomics profiling, we validated these findings in rhesus macaques challenged with SARS-CoV-2 WA1/2020 or SARS-CoV-2 B.1.351. Finally, we show that Ad26.COV2.S vaccination induces T and B cell signatures that correlate with binding and neutralizing antibody responses weeks following vaccination. These data provide insights into the molecular mechanisms of Ad26.COV2.S protection against severe COVID-19 in animal models.

## Author summary

In this study, we show that vaccination with Ad26.COV2.S protected hamsters and rhesus macaques challenged with SARS-CoV-2 from developing excessive proinflammatory responses mediated by IL-6 and IL-1, macrophages, and neutrophil activation. Ad26 vaccination also prevented the upregulation of coagulation and clotting cascades pathways. In contrast, unvaccinated challenged animals showed a significant increase of these proinflammatory and prothrombotic pathways. These data provide insights into the molecular mechanisms of Ad26.COV2.S protection against SARS-CoV-2.

## Introduction

The COVID-19 pandemic has sparked intense interest in the rapid development of vaccines and animal models to evaluate vaccine candidates and define molecular and immunologic correlates of protection. We and others have reported that animal models such as rhesus macaques and hamsters can be infected with SARS-CoV-2 WA1/2020 and variants of concern and show robust viral replication in the upper and lower respiratory tract, enabling studies of COVID-19 pathogenesis and prevention with vaccines in these animal models [1–5].

Syrian golden hamsters show productive viral replication, lung pathology, and mortality when challenged with SARS-CoV2 [3,6–8], making them pertinent for vaccine evaluation. Reports of transcriptomics and proteomics profiling of blood and lung tissues from hamsters infected with SARS-CoV-2 have shown significant upregulation of interferon and proinflammatory pathways, activation of the complement system, and recruitment of neutrophils and macrophages to the lung of infected hamsters that correlate with the presence of SARS-CoV-2 viral RNA [6,9], supporting the role of these pro-inflammatory responses in COVID-19 severity [10]. It is essential to assess whether vaccines modulate these host immune and transcriptional responses and protect from excessive pro-inflammatory responses induced by SARS-CoV-2.

Ad26.COV2.S is a replication-incompetent human adenovirus type 26 vector [11] that expresses a prefusion stabilized SARS-CoV-2 spike protein (S) [12,13] from the Wuhan 2019 strain of SARS-CoV-2. We previously reported that Ad26.COV2.S demonstrated protective efficacy against SARS-CoV-2 challenge in hamsters and nonhuman primates [3,14–16] and showed safety and immunogenicity in humans [17]. A phase III efficacy trial has been demonstrated that Ad26.COV2.S provided 86%, 88%, and 82% protection against severe COVID-19 disease by day 28 after vaccination in the USA, Brazil, and South Africa, respectively [17]. In rhesus macaques, we also showed Ad26.COV2.S provided robust protection against the B.1.351 challenge [16].

In this study, we performed in-depth analyses of bulk RNA-Seq transcriptomic profiling of lung tissues at day 4 post- SARS-CoV-2 challenge from Ad26.COV2.S vaccinated and sham unvaccinated hamsters. To characterize Ad26 vaccine-mediated protection from severe COVID-19 in hamsters, we integrated the transcriptomics data with virological data as well as adaptive immune responses elicited by Ad26 at weeks 2 and 4 following immunization. Vaccination with Ad26.COV2.S (also termed Ad26.S.PP) attenuated the upregulation of proinflammatory pathways and prevented the upregulation of the coagulation cascade pathways such as platelet aggregation, blood coagulation, and the clotting cascade. We also demonstrated that Ad26.CoV2.S protects against SARS-CoV-2 WA1/2020 and B1.351 induced-pathological pathways using proteomics profiling in rhesus macaques. Together, these results provide new insights into the molecular and immunological mechanisms of Ad26.COV2.S protection from SARS-CoV-2.

## Results

### Study design

We recently reported a study in which recombinant, replication-incompetent Ad26 vectors expressing SARS-CoV-2 Spike constructs prevented clinical disease in Syrian golden hamsters after challenge [3]. We studied two SARS-CoV-2 Spike constructs administered at either 10^10^ or 10^9^ viral particles (vp) and a sham control (5 total groups, n = 10 per group). One Spike construct encoded a deletion of the transmembrane region and cytoplasmic tail reflecting the soluble ectodomain with a foldon trimerization domain (Ad26.S.dTM.PP), while the other encoded the full-length spike (Ad26.S.PP; renamed Ad26.COV2.S for clinical development). Both constructs contained two stabilizing proline mutations at the furin cleavage site. At 4 weeks, all 50 hamsters were challenged with 5.0 × 10^5^ TCID50 of the USA-WA1/2020 strain. While both constructs prevented severe weight loss, we observed that the Ad26.S.PP construct exhibited superior immunogenicity and protection [3]. To gain insights into the molecular mechanisms of severe COVID-19 and Ad26-mediated protection in Syrian golden hamsters, we euthanized a subset of these animals at 4 days post-infection (4 dpi) with SARS-CoV-2. We performed bulk RNA sequencing (RNA-seq) from lung tissues. Specifically, we studied three sham unvaccinated hamsters, four Ad26.S.dTM.PP-vaccinated hamsters (two at 10^10^ and two at 10^9^), and five Ad26.S.PP-vaccinated hamsters (two at 10^10^ and three at 10^9^). For comparison, we additionally obtained lung tissues from three naïve animals that were neither vaccinated with Ad26 nor challenged with SARS-CoV-2 (**S1A Fig**).

### Ad26 vaccination eliminates detection of SARS-CoV-2 reads by bulk RNA-Seq

**Fig 1:**
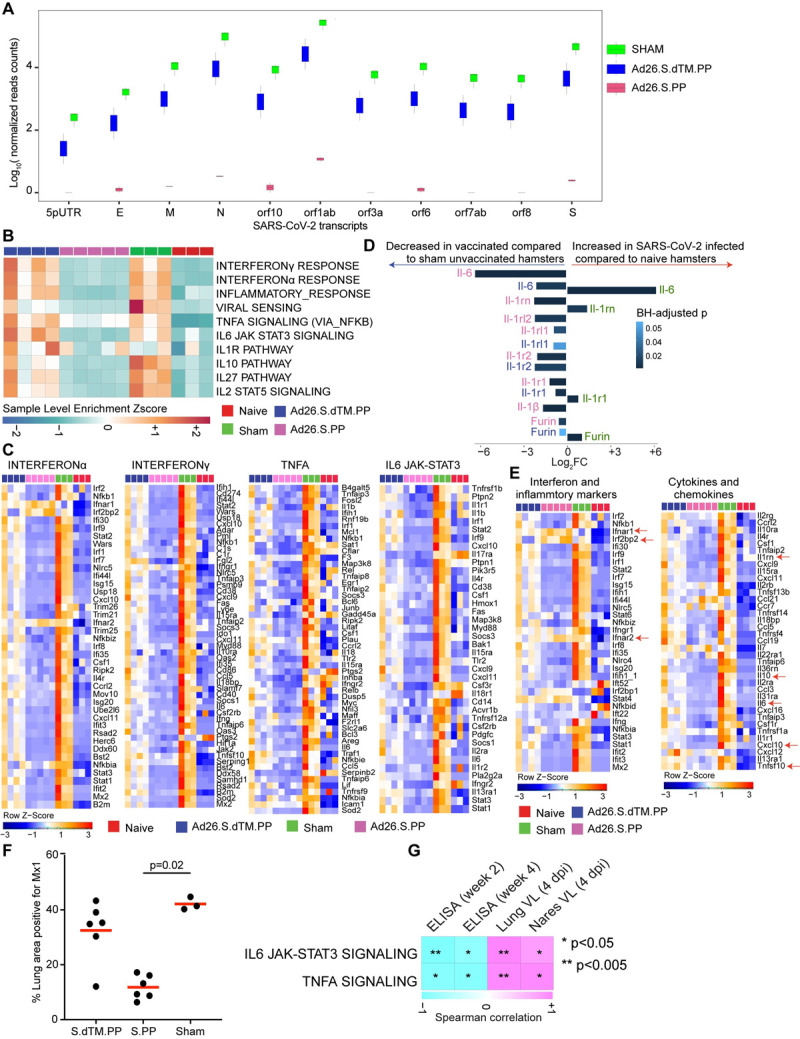
Proinflammatory signaling pathways were decreased in Ad26.COV2.S vaccinated compared to sham unvaccinated hamsters. **(A)** Boxplots of SARS-CoV-2 transcripts normalized read counts across all animals, vaccinated hamsters are shown in blue and pink, and sham animals in green. **(B)** Sample-enrichment analysis (SLEA) of inflammatory pathways and interferon signaling increased by SARS-CoV-2 at 4 dpi in sham unvaccinated hamsters (in green) and decreased in vaccinated hamsters (in pink and blue) compared to naïve uninfected animals (in red). Heatmap presenting the SLEA z-score of each of these pathways. SLEA represents the mean expression of all significant genes within each pathway for each animal. An SLEA z-score greater than 0 corresponds to a pathway for which member genes are up-regulated, while an SLEA z-score inferior to 0 corresponds to a pathway with genes downregulated in that sample. Columns correspond to individual animals, and rows correspond to individual pathways scaled across all animals using the z-score R function. **(C)** Gene heatmaps of the row scaled expression of the leading genes from pathways shown in panel A increased or decreased at 4 dpi in vaccinated hamsters compared to sham unvaccinated and control naïve animals. Adjusted P-values (<0.05) were calculated by DEseq2 using Benjamini-Hochberg corrections of Wald test p-values. Columns correspond to individual animals, and rows correspond to individual genes. **(D)** Bar plots represent the log2 fold change mRNA expression of IL-6, IL-1, IL-1R, and Furin across all groups. The length of the bar plot represents the log_2_-fold change compared to control animals, and the color gradient corresponds to the BH-adjusted p-values for each gene. **(E)** Heatmaps of proinflammatory markers and proinflammatory cytokines at 4 dpi in vaccinated hamsters compared to sham unvaccinated and control naïve animals. Adjusted P-values (<0.05) were calculated by DEseq2 using Benjamini-Hochberg corrections of Wald test p-values. Columns correspond to individual animals, and rows correspond to individual genes scaled across all animals. **(F)** Percent area of lung tissue staining positive for Mx1. Mx1 = myxovirus protein 1 (type 1 interferon-inducible gene) in vaccinated compared to sham unvaccinated hamsters. Kruskall-Wallis one-way ANOVA followed by Dunn’s multiple comparisons test. Red line = median. **(G)** Correlation matrix plot showing the Spearman correlation of interferon and inflammatory signatures at 4 dpi with viral loads in hamster’s lung and nares at 4 dpi and neutralizing and binding antibody titers at weeks 2–4 post-vaccination. Negative correlations were shown in cyan, and positive correlations were shown in pink.

Transcriptomic profiling using bulk RNA-Seq analysis showed that the overall transcriptomic profiles of animals within each group of hamsters were homogenous, with no significant differences between groups of animals that received different vaccine doses (**S1B and S1C Fig**). We mapped the RNA-Seq reads to the SARS-CoV-2 genome and observed a significant number of reads that mapped to SARS-CoV-2 transcripts, ranging from 300 to 315000 reads, in the sham unvaccinated hamsters. However, fewer reads were mapped to SARS-CoV-2 transcripts in the S.dTM.PP-vaccinated hamsters, ranging from 10 to 16000 reads (**Fig 1A and S1 Table**). In contrast, only a very small fraction of SARS-CoV-2 reads were detected in the Ad26.COV2.S vaccinated hamsters with only 0–12 reads mapping SARS-CoV-2 transcripts. We observed a significant reduction in SARS-CoV-2 reads in the Ad26.COV2.S inoculated hamsters compared to the sham unvaccinated animals (**Fig 1A**). Additionally, the normalized reads count of SARS-CoV-2 transcripts correlated positively with viral loads in the lung and nares of vaccinated hamsters (**S2A and S2B Fig**).

We previously showed that detection of SARS-CoV-2 Envelope gene subgenomic RNA (sgRNA) by quantitative polymerase chain reaction (qPCR) was markedly diminished by Ad26 vaccination [3], validating our RNA-seq data. Differential expression gene analysis (DEGs) showed differences in upregulated or downregulated genes in vaccinated and sham unvaccinated animals compared to naïve animals (**S3A Fig**). Moreover, we observed significant differences in the transcriptomic profile of hamsters vaccinated with the Ad26.COV2.S compared to the sham unvaccinated animals with 3401 differentially expressed genes between the two groups, whereas only 87 genes were differentially expressed between the Ad26.S.dTM.PP vaccinated hamsters compared to sham-unvaccinated animals (**S3B Fig**) (Wald test, BH-adjust P<0.05). Moreover, we observed downregulation of pro-inflammatory markers such as Mx2, Irf3, Tlr7, Cxcl10, and Cd68 in the Ad26.COV2.S compared to the Ad26.S.dTM.PP vaccinated hamsters (**S3C Fig).** The expression of interferon receptors, Ifnar1 and Ifnar2, and interferon regulatory factor 2-binding protein 2 (Irf2bp2) were higher in vaccinated hamsters compared to sham unvaccinated and naïve animals (**S3D Fig).** These data support the superior protection observed with Ad26.S.PP [3] and suggest that interferon alpha contribute to the Ad26.COV2.S protection from severe disease. Detection of SARS-CoV-2 transcripts in the lung of sham unvaccinated hamsters is consistent with our previous report showing that sham unvaccinated hamsters showed robust viral replication in the upper and lower respiratory tract and experienced more weight loss, extensive pneumonia, and mortality compared to hamsters immunized with Ad26.COV2.S vaccine [3].

### Ad26.COV2.S vaccination attenuates interferon and inflammatory signaling pathways following SARS-CoV-2 challenge

Excessive inflammatory response to SARS- CoV-2 is a major cause of disease severity and death in COVID-19. Proinflammatory cytokines and chemokines were previously reported in blood and lung tissue of hamsters infected with SARS-CoV-2 and in COVID-19 patients [6,18,19] (**S4A and S4B Fig and S2 Table**). We interrogated our transcriptomic data in vaccinated hamsters compared to naïve and sham groups for pro-inflammatory pathways that are central for the pathogenesis of severe COVID-19 [10,20–22]. Gene set enrichment analysis (GSEA) of DEGs at 4 dpi showed pathways of interferon regulatory or stimulated genes, inflammasome, and proinflammatory cytokines signaling such as interferon-alpha, TNF, IL-1, and IL-6 signaling pathways, were significantly increased in sham unvaccinated compared to naïve hamsters as shown by the positive pathway normalized enrichment score (NES) (interferon-alpha (NES = 2.74, FDR value <10^−6^); TNFA (NES = 2.34, FDR <10^−6^); IL6_JAK-STAT3 (NES = 2.31, value <10^−6^); INFLAMMATORY RESPONSE (NES = 2.39, FDR <10^−6^); IL1R signaling (NES = 1.44, FDR = 0.07)) as shown by the pathways individual score (sample level enrichment analysis: SLEA score) across all animals (**Fig 1B and 1C**). A direct comparison between vaccinated and sham unvaccinated hamsters at 4 dpi revealed a significant decrease of proinflammatory pathways in the Ad26.COV2.S vaccinated compared to sham unvaccinated animals (interferon-alpha (NES = -2.08, FDR<10^−6^); TNFA (NES = -2.47, FDR<10^−6^), IL6_JAK-STAT3 (NES = -2.41, FDR<10^−6^); INFLAMMATORY RESPONSE (NES = -2.55, FDR<10^−6^); IL1R signaling (NES = -1.58, FDR = 0.03)) (**Fig 1B and 1C**). Further, the expression of major proinflammatory cytokines and chemokines that contribute to SARS-CoV-2 pathogenesis, such as Il-6, Il-1α, Il-1β, Stat1/2/3, Furin, IL-2rg, Ccl21, Cxcl10, Csf, and Tnf were significantly increased in sham unvaccinated (shown in green) compared to naïve control (shown in red) hamsters (Wald test, BH-adjust P<0.05) (**Fig 1D and 1E)**. Whereas the mRNA expression levels of these pro-inflammatory markers are comparable between vaccinated and naïve animals (**Fig 1E)**.

Using immunohistochemistry (IHC), we showed a significant reduction of the interferon-inducible protein Mx1 in the lung of Ad26.COV2.S vaccinated hamsters compared to sham-infected unvaccinated hamsters (p = 0.02) (**Fig 1F)**. Furthermore, we observed that IL6_JAK-STAT3 and TNF alpha signaling proinflammatory pathways correlated negatively with Ad26 elicited ELISA binding and neutralizing antibody titers [3] at weeks 2–4 following immunization (**Figs 1G and S5)**. In contrast, these pathways correlated positively with viral loads in the lung and nares of vaccinated hamsters (**Figs 1G and S5)**.

### Ad26.COV2.S vaccination attenuates upregulation of pathways of macrophage activation, monocytes, and neutrophils

**Fig 2:**
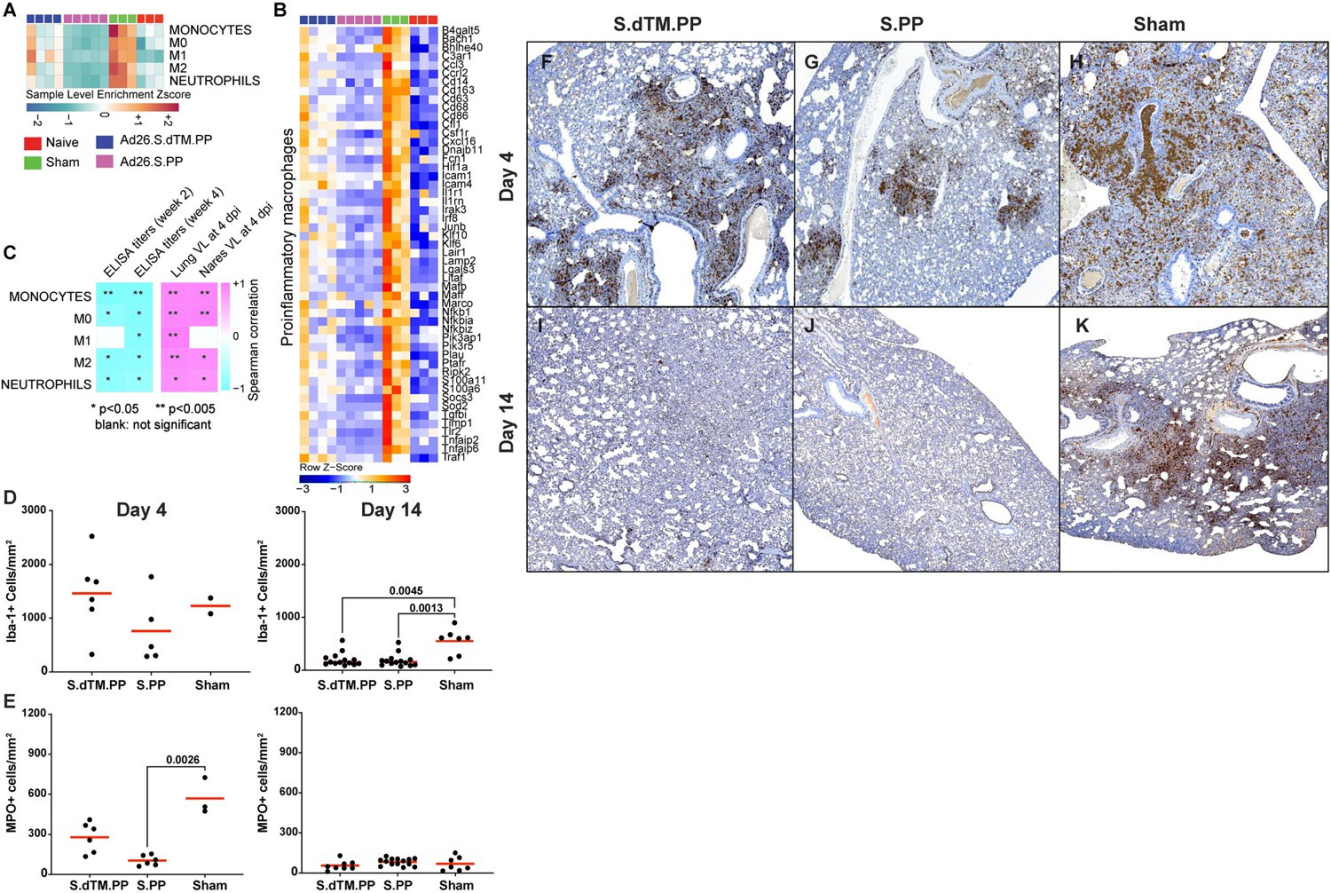
Pathways of macrophages and neutrophils were decreased in Ad26.COV2.S vaccinated compared to sham unvaccinated hamsters. **(A)** Sample-enrichment analysis (SLEA) of pathways of monocytes, macrophages M1 and M2 pathways, and neutrophils signaling at 4 dpi following SARS-CoV-2 challenge in vaccinated hamsters (shown in pink and blue colors), sham unvaccinated hamsters (shown in green) and in naïve control hamsters (shown in red). Heatmap presenting the SLEA z-score of each of these pathways. SLEA scores represent the mean expression of all significant genes within each pathway for each animal. An SLEA z-score greater than 0 corresponds to a pathway for which member genes are up-regulated, while an SLEA z-score inferior to 0 corresponds to a pathway with genes downregulated in that sample. Columns correspond to individual animals, and rows correspond to individual pathways scaled across all animals using the z-score R function. **(B)** Heatmaps of the normalized expression of the leading genes contributing to the enrichment of proinflammatory macrophages increased by SARS-CoV-2 in sham animals (in green) and decreased in Ad26 vaccinated hamsters (in pink and blue) at 4 dpi. **(C**) Correlation matrix plot showing the Spearman correlations of the SLEA z-score of monocytes, macrophages (M0, M1and M2), and neutrophils pathways at 4 dpi with viral loads in hamster’s lung and nares at 4 dpi, and with neutralizing and binding antibody titers at weeks 2–4 post-vaccination in vaccinated hamsters. Negative correlations were shown in cyan, and positive correlations were shown in pink. (**D-K**) Myeloid inflammation resolves by 14 days following challenge in vaccinated hamsters. Immunohistochemistry (IHC) was performed on lung specimens from WA1/2020 challenged Syrian hamsters for macrophages (Iba-1) and neutrophils (myeloperoxidase, MPO) at 4 (**D, E** left panels) and 14 days (**D, E** right panels) following challenge and cells per unit area were calculated using quantitative image analysis. (**F-K**) Representative images of Iba-1 (IHC) from S.dTM.PP (**F, I**), S.PP (**G, J**), and sham (**H, K**) hamsters 4 days and 14 days following challenge. Kruskall-Wallis one-way ANOVA followed by Dunn’s multiple comparisons test. Red line = median.

Previous bulk and single-cell transcriptomics and proteomics studies in COVID-19 patients, macaques, and hamsters infected with SARS-CoV-2 showed upregulation of macrophage and neutrophils signature that correlated with disease severity [4,6,10,19,21–23]. Consistent with these reports, we observed that pathways of monocyte (NES = 1.79, FDR = 0.006), macrophage M1(NES = 2.41, FDR<10^−6^), and M2 (NES = 1.86, FDR = 0.008), and neutrophil (NES = 1.58, FDR = 0.04) signaling were significantly increased in sham unvaccinated hamsters compared to naïve animals (**Fig 2A**). Vaccination with Ad26 prevented the upregulation of these pathways in the Ad26.COV2.S group at 4 dpi (**Fig 2A and 2B**). Further, we observed that markers of proinflammatory macrophages, including Ccl3, Cd163, Cd68, Csf1r, and Marco [6], were significantly increased in sham unvaccinated hamsters compared to naïve animals, while in the Ad26.COV2.S vaccinated hamsters, the expression of these macrophages markers was comparable to naïve animals (**Fig 2B).** Further, transcriptomic signatures of monocytes, macrophages, and neutrophils correlated negatively with Ad26-induced binding and neutralizing titers at weeks 2–4 but were positively correlated with viral loads in the lung and nares of vaccinated hamsters (**Figs 2C and S6**). Using histopathology analysis, we showed that vaccination with Ad26.COV2.S reduced myeloperoxidase expressing myeloid cells, most of which are neutrophils with Ad26.COV2.S vaccinated animals showed significantly lower neutrophils than sham vaccinated animals at days 4 and 14 (**Fig 2D and 2E**). Macrophage and neutrophil inflammation largely resolved in vaccinated animals by 14 days following challenge (**Fig 2F, 2G, 2I and 2J**), however sham animals had persistently higher numbers of macrophages compared to vaccinated animals and persisted at day 14 (**Fig 2H and 2K**).

### Ad26.COV2.S vaccination attenuates signatures associated with complement activation and coagulation cascades

**Fig 3:**
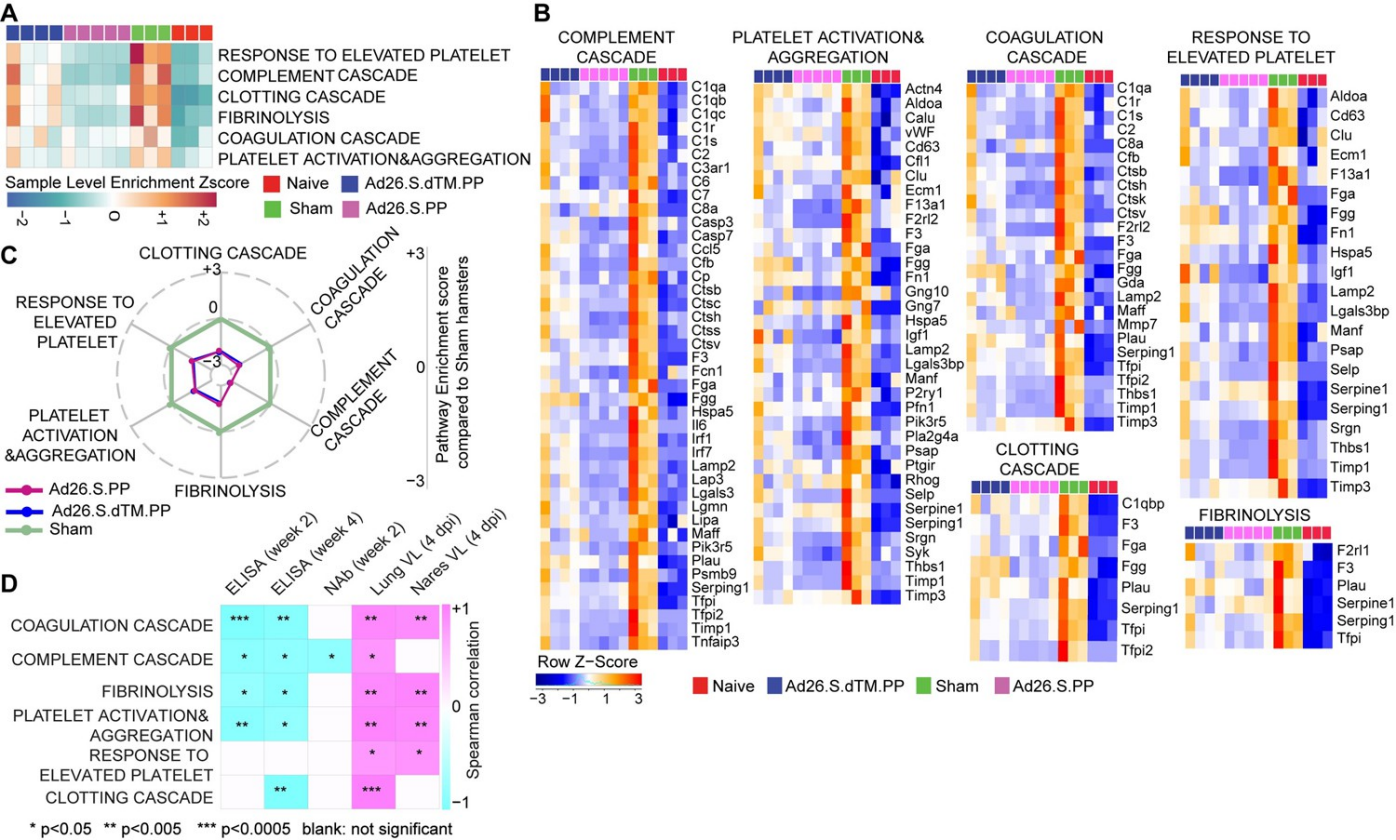
Ad26.COV2.S vaccination attenuates signatures of complement system activation and coagulation cascade associated with severe COVID-19. **(A)** Sample-enrichment analysis (SLEA) of pathways of thrombosis-associated pathways decreased in Ad26 vaccinated animals at 4 dpi (in pink and blue) compared to sham-unvaccinated animals (in green). Heatmap presenting the SLEA z-score of each of these pathways. SLEA scores represent the mean expression of all significant genes within each pathway for each animal. **(B)** Heatmaps of the normalized expression of the leading genes contributing to the enrichment of thrombosis-associated pathways increased by SARS-CoV-2 in sham animals (in green) and decreased in Ad26 vaccinated hamsters (in pink and blue) at 4 dpi. **(C)** Radar plot showing the GSEA normalized enrichment score (NES) of thrombosis-associated pathways decreased in Ad26 vaccinated hamsters in blue and pink compared to sham unvaccinated animals in green. **(D)** Correlation matrix plot showing the Spearman correlations of thrombosis-associated pathways at 4 dpi with viral loads in hamster’s lung and nares at 4 dpi and neutralizing and binding antibody titers at weeks 2–4 post-vaccination. Negative correlations were shown in cyan, and positive correlations were shown in pink.

We next examined the effect of Ad26 vaccination on additional pathways known to play prominent roles in the pathogenesis of severe COVID-19, including activation of complement and coagulation cascades [19,24,25]. Unvaccinated sham hamsters showed significant upregulation of pathways of clotting cascade (NES = 1.93, FDR = 0.001), coagulation cascade (NES = 1.66, FDR = 0.02), complement activation (NES = 2.35, FDR<10^−6^), platelet activation and aggregation (NES = 2.07, FDR = 0.0002) and fibrinolysis (NES = 2.07, FDR<10^−6^) compared to naïve animals as shown by the sample level enrichment analysis (SLEA) for each individual animal (**Fig 3A).** Markers of these SARS-CoV-2 induced pathways in sham hamsters were enriched in complement components C3, C7, C2; clotting and coagulation cascade and tissue factors F3, F5, Plau, Fga; and platelet activation and aggregation markers such as Clu, Timp1, Thbs1, Sh2b2, and Vav1 (**Fig 3B).** These markers were not significantly increased in the Ad26 vaccinated hamsters (**Fig 3B).** When compared to sham unvaccinated, vaccinated hamsters showed significant downregulation of pathways of clotting cascade (NES = -1.98, FDR = 0.001), coagulation cascade (NES = -2.23, FDR<10^−6^), complement activation (NES = -2.04, FDR = 0.0007), platelet activation and aggregation (NES = -1.64, FDR = 0.02) and fibrinolysis (NES = -1.5, FDR = 0.05) (**Fig 3C**), suggesting Ad26 vaccination protected hamsters from activation of the clotting and the coagulation cascades. Moreover, we found that pathways of the complement cascade, platelet activation and aggregation, coagulation cascade, and fibrinolysis correlated negatively with weeks 2–4 ELISA binding and neutralizing antibody titers elicited by Ad26 vaccination and positively correlated with 4 dpi viral loads in lung and nares of vaccinated hamsters (**Figs 3D and S7)**.

### Ad26.COV2.S vaccination attenuates signatures of proinflammatory pathways and coagulation cascades in rhesus macaques challenged with SARS-CoV-2 WA1/2020 or B.1.351

**Fig 4:**
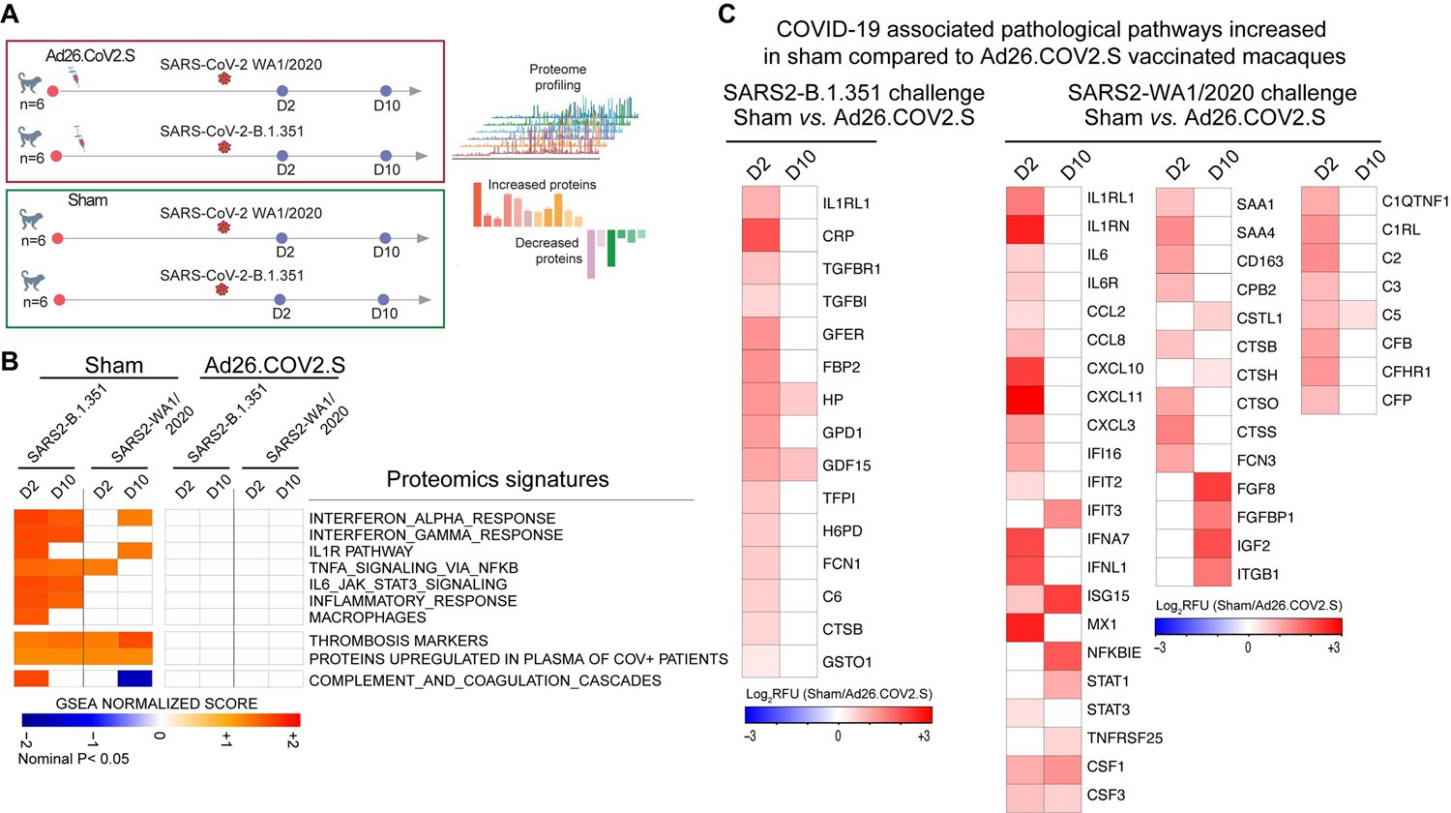
Ad26.COV2.S vaccination attenuates proteomics signatures of inflammation and thrombosis pathways in rhesus macaques challenged with SARS2 WA1/2020 or B.1.351. (**A**) Study design. (**B**) Heatmap representation of the GSEA normalized enrichment score (NES) of pathways of inflammation, thrombosis, and the coagulation cascades in sham or Ad26.COV2.S vaccinated macaques at days 2 and 10 following challenge with WA1/2020 or with B.1.351. NES scores raging from blue (decreased), white (not significant), or red (increased) in sham unvaccinated compared to vaccinated macaques. (**C**) Heatmap representation of the log2 fold change of selected increased proteins in serum of sham unvaccinated compared with vaccinated macaques, for macaques challenged with the B.1.351 variant (left panel) or with the WA1/2020 challenge (right panel). Values represent the relative fluorescence unit (RFU) levels of each individual protein, ranging from blue (decreased), white (not significant), or red (increased).

To further explore the mechanisms of Ad26.COV2.S protective efficacy against pathological pathways induced by SARS-CoV-2, we collected serum samples from rhesus macaques vaccinated with the Ad26.COV2.S and challenged with SARS-CoV-2 WA1/2020 or B.1.351 [16]. For comparison, we included two unvaccinated sham groups challenged with SARS-CoV-2 WA1/2020 or B.1.351 (**Fig 4A**). The immunological and virological data from this macaque study [16] and showed that Ad26.COV2.S provided robust protection against WA1/2020 and B.1.351 in rhesus macaques.

We performed proteomics profiling using the SomaScan 7K platform. Enrichment analysis showed that pathways of interferon and proinflammatory signaling, IL1, TNF, and IL6 pathways were significantly increased (GSEA: nominal p<0.05) in sham unvaccinated macaques challenged with WA1/2020 or B.1.351 at day 2 following challenge and persisted at day 10 post-challenge in sham unvaccinated macaques (**Fig 4B**). In contrast, proinflammatory pathways of IL6, IL1, and TNFA signaling were not increased in Ad26.COV2.S vaccinated macaques (**Fig 4B**). Further, proteomics analysis showed that pathways of thrombosis and markers upregulated in plasma from severe COVID-19 patients [26] were upregulated in sham macaques challenged with WA1/2020 or B.1.351 variant but were unchanged in vaccinated animals (**Fig 4B**).

Although we observed differences in the innate proinflammatory programs induced by the WA1/2020 or the B.1.351 challenge, serum levels of significant proinflammatory markers associated with COVID-19 severity, such as IL6 and IL1, CCL8 NFKB, STAT3, MX1, and CSF, were significantly increased in sham unvaccinated compared with vaccinated macaques (**Fig 4C).** Similarly, markers of the complement cascade (C6, C2, C3, CFB), clotting, and coagulation cascades (TFPI, FBP2, TGFB, FCN1) were increased in sham unvaccinated compared to vaccinated macaques at days 2–10 following challenge **(Fig 4C)**. Together, our data show that Ad26.COV2.S vaccination in macaques protects from excessive inflammatory responses and pathological pathways of thrombosis and the coagulation cascade induced by SARS-CoV-2 WA1/2020 or B.1.351. Additional studies are warranted to investigate the innate immune programs induced by different SARS-CoV-2 variants of concern and how COVID-19 vaccines modulated these programs.

### Ad26 vaccination induces T and B cell signatures that correlated with Ad26 induced humoral immune responses

**Fig 5:**
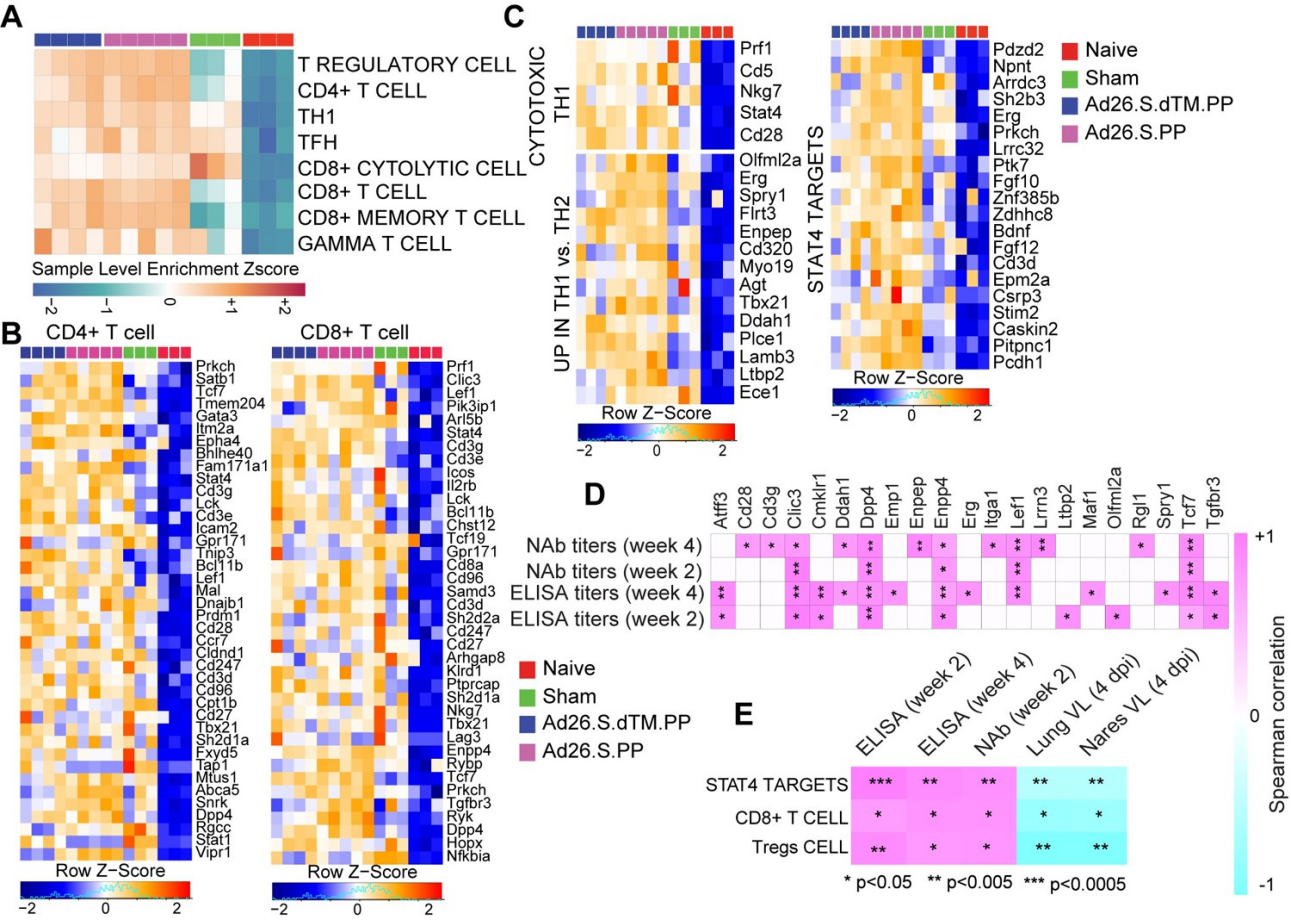
Ad26.COV2.S vaccination increased signatures of CD8+ and CD4+ T cell in vaccinated hamsters. **(A)** Sample-enrichment analysis (SLEA) of pathways of signatures of CD4, CD8, T regulatory cell (Tregs), T follicular helper cell (TFH) and T helper 1 (Th1) cell increased in vaccinated animals at 4 dpi (in pink and blue) compared to naïve and sham animals (in red and green). Heatmap presenting the SLEA z-score of each of these pathways. SLEA scores represent the mean expression of all significant genes within each pathway for each animal. An SLEA z-score greater than 0 corresponds to a pathway for which member genes are up-regulated, while an SLEA z-score inferior to 0 corresponds to a pathway with genes downregulated in that sample. Columns correspond to individual animals, and rows correspond to individual pathways scaled across all animals using the z-score R function. **(B-C)** Heatmaps of the leading genes of CD8+, CD4+, Tregs, and Th1 markers, increased at 4 dpi in vaccinated animals compared to naïve animals. Adjusted P-values (<0.05) were calculated by DEseq2 using Benjamini-Hochberg corrections of Wald test p-values. Columns correspond to individual animals, and rows correspond to individual genes normalized reads count scaled across all animals. **(D-E)** Matrix showing the Spearman correlations of CD4 T cell markers (D) and CD8 T cell signature, Tregs signature, and STAT4 targets (E) at 4 dpi with viral loads in hamster’s lung and nares, and with neutralizing and binding antibody titers at weeks 2–4 in vaccinated hamsters. Negative correlations were shown in cyan, and positive correlations were shown in pink.

We next investigated whether Ad26 vaccination-induced molecular signatures are consistent with protective adaptive immune mechanisms observed in SARS-CoV-2 infected humans, macaques and hamsters vaccinated with Ad26.COV2.S [3,14,16,27]. GSEA showed that signatures of CD4+ T cell (NES = 2.27, FDR<10^−6^), CD8+ T cell (NES = 1.73, FDR = 0.02), T helper follicular cell (Tfh) (NES = 1.83, FDR = 0.009), and T regulatory cell (NES = 2.07, FDR = 0.0003), were enriched in Ad26-vaccinated hamsters compared to naïve controls (**Fig 5A and 5B)**. GO term analysis of CD4+ T cell pathway (Cd28, Cd3g, Cd3e, Cd3d, Lck, Fyn, Cd247, Ccr7, Dpp4, Icos), showed enrichment of markers of lymphocyte co-stimulation (FDR = 2.65 x 10^−12^), leukocyte activation (FDR = 7.25 x10^-12^), and T cell activation (FDR = 1.18 x 10^−10^). Moreover, key markers of cytotoxic CD8+ T cell adhesion, activation, and effector memory functions such as Prf1, Lef1, Lck, Tcf7, and Nkg7, were increased by Ad26.COV2.S in vaccinated compared to naïve control animals (**Fig 5B**), suggesting CD8+ T cell-induced by Ad26 are engaged in the recognition and elimination of infected cells [28]. Similarly, Th1 cell signatures were significantly upregulated in Ad26-vaccinated compared to naïve control animals and included Th1 cytotoxic genes (NES = 2.01, FDR<10^−6^), genes increased in Th1 cells (NES = 1.6, FDR = 0.02) and STAT4 signaling, a major transcription factor involved in Th1 differentiation and proliferation [29] (NES = 1.31, FDR = 0.02) (**Fig 5C)**. Further, we observed that signature of pathological T regulatory cell (Tregs) increased in patients with severe COVID-19 disease [30], was increased in sham unvaccinated compared with Ad26.COV2.S vaccinated hamsters (**S8 Fig**).

We correlated the expression of T cell transcriptomic signatures induced in vaccinated hamsters with humoral immune responses elicited by the Ad26.COV2.S vaccine at weeks 2–4. We observed a significant positive correlation of markers of CD4+ T cell (**Fig 5D**), signatures of CD8+ T (**Figs 5E and S9A**) cell and T regulatory cell signatures with ELISA, and neutralizing antibody titers at weeks 2 and 4. In contrast, these signatures correlated negatively with viral loads in the lung and nares of vaccinated hamsters (**Fig 5E**). Similarly, we found that STAT4 targets, a major transcription factor involved the Th1 activation [29,31], were positively correlated with ELISA and neutralizing antibody titers at weeks 2 and 4 and negatively correlated with viral loads in the lung and nares of vaccinated hamsters (**Figs 5E and S9A)**.

**Fig 6:**
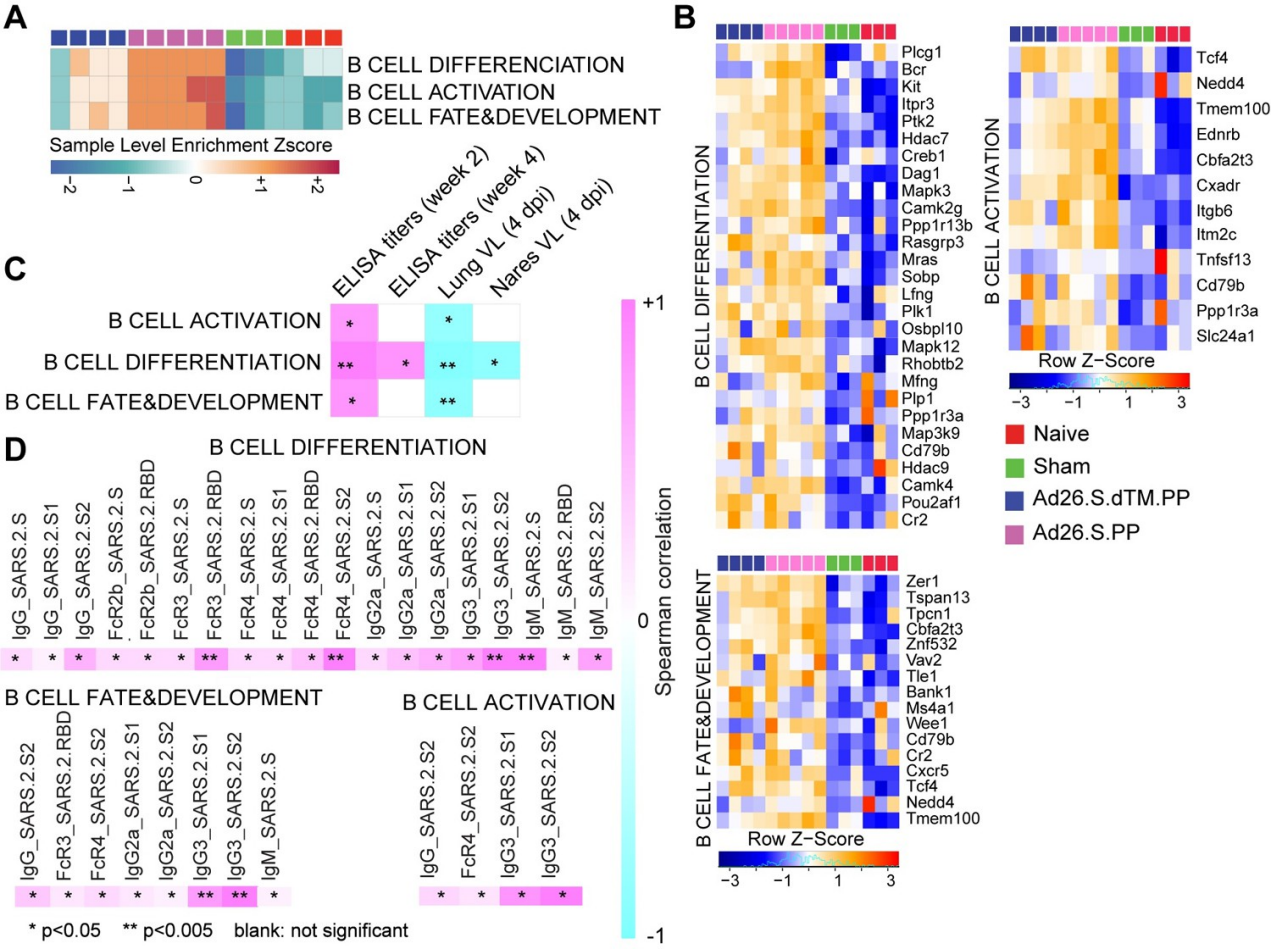
Pathways of B cell increased in Ad26.COV2.S vaccinated compared to naïve and sham hamsters. **(A**) Sample-enrichment analysis (SLEA) of pathways of B cell activation, differentiation and B cell fate increased in vaccinated animals at 4 dpi (in pink and blue) compared to naïve and sham animals (in red and green). Heatmap presenting the SLEA z-score of each of these pathways. SLEA represents the mean expression of all significant genes within each pathway for each animal. **(B**) Heatmaps of the leading genes of B cell markers from pathways shown in the top panel increased at 4 dpi in vaccinated compared to sham and naïve hamsters. Adjusted P-values (<0.05) were calculated by DEseq2 using Benjamini-Hochberg corrections of Wald test p-values. Columns correspond to individual animals, and rows correspond to particular gene normalized reads count scaled across all animals. **(C)** Matrix showing the spearman correlation of B cell signatures at 4 dpi with viral loads in hamster’s lung and nares, and with neutralizing and binding antibody titers at weeks 2–4 in vaccinated hamsters. Negative correlations were shown in cyan, and positive correlations were shown in pink. **(D)** Matrix showing the Spearman correlations of B cell pathways with S-specific and RBD-specific antibody responses in the Ad26.COV2.S vaccinated animals at week 4 by systems serology, including IgG, IgG2a, IgG3, IgM, Fc-receptors FcRγ2, FcRγ3, FcRγ4, and antibody-dependent complement deposition (ADCD). All positive correlations were shown in pink. Correlations were assessed using Spearman correlation.

Given the critical role of humoral immune responses in immunity to SARS-CoV-2 infection [28], we next characterized B cell responses following Ad26 vaccination in hamsters. We observed a significant increase in signatures of B cell activation and differentiation and other markers regulating B cell fate and development in vaccinated hamsters compared to sham and naïve animals, as shown by the SLEA scores in each animal (**Fig 6A**) and by the upregulation of the top markers of B cell activation and differentiation and markers regulating B cell fate and development such as Cd79, Pou2af1, Bank1and Tcf4 (**Fig 6B**). These pathways correlated positively with binding and neutralizing antibody titers elicited by Ad26 at weeks 2 and 4 (**Figs 6C and S9B**) but were correlated negatively with viral loads in the lung and nares of vaccinated hamsters (**Fig 6C**). In macaques, proteomics pathways of BCR signaling and antigen presentation correlated positively with binding and neutralizing titers (**S10B Fig).**

Additionally, pathways of B cell activation, differentiation, and development correlated positively with S-specific and RBD-specific antibody IgG, IgG2a, IgG3, IgM, Fc-receptors FcRγ2, FcRγ3, and FcRγ4 and antibody-dependent complement deposition (ADCD) responses in vaccinated animals at week 4 assessed by systems serology and previously published by our group [3] (**Fig 6D**).

## Discussion

**Fig 7:**
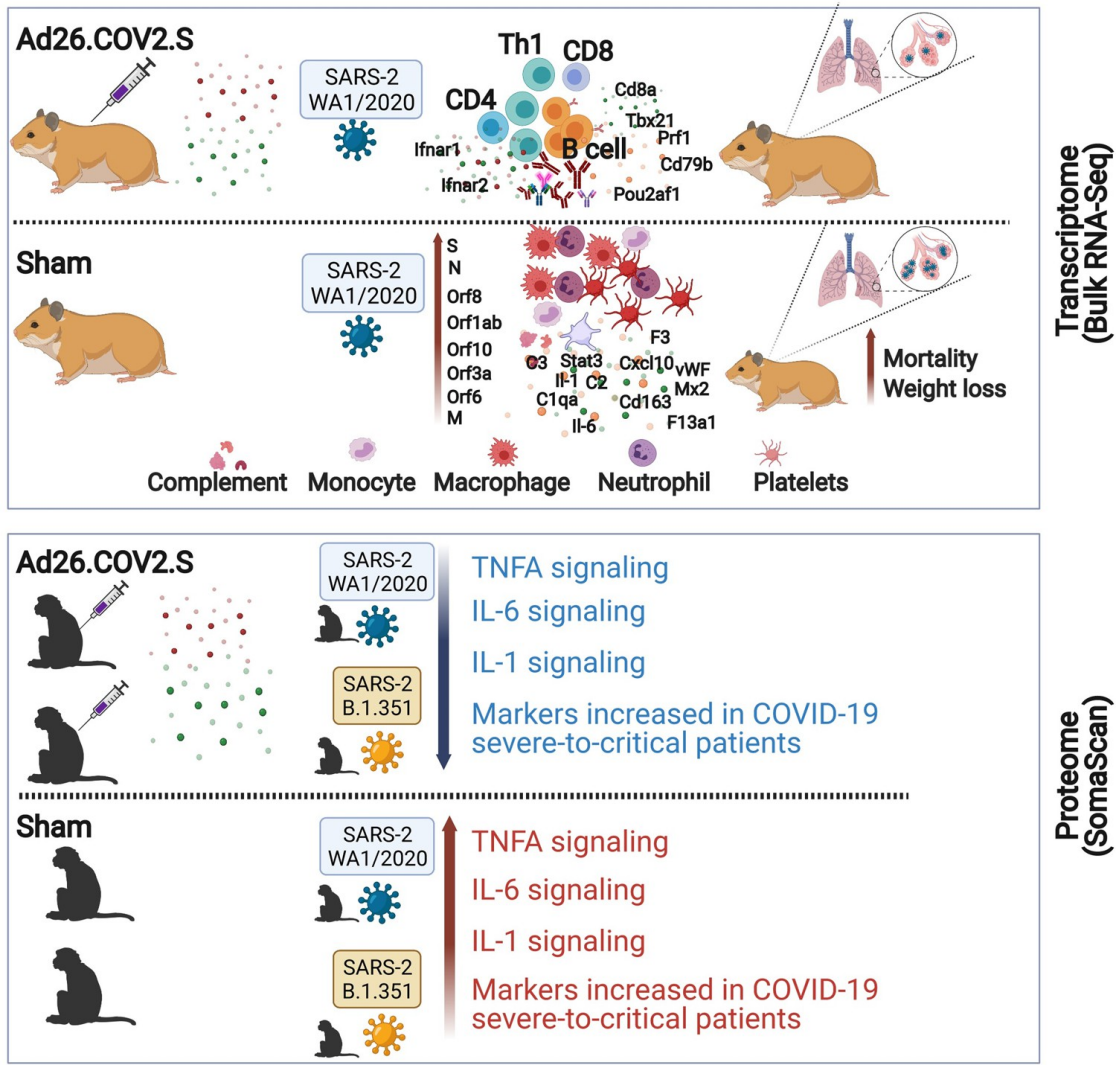
Ad26.COV2.S abrogates proinflammatory and thrombosis associated pathways in hamsters and macaques challenged with SARS-CoV-2 WA and B.1.351. Fig 7 was created with BioRender.com.

We previously showed that vaccination with Ad26.COV2.S, an Ad26 vector encoding a full-length prefusion stabilized S immunogen (S.PP) protected against SARS-CoV-2 challenge in hamsters [3] and macaques [14]. In this study, we used bulk RNA-Seq in hamsters and serum proteomic profiling in rhesus macaques and showed that Ad26.COV2.S attenuates COVID-19 hallmark pathological pathways (**Fig 7**).

Proinflammatory pathways of IL6, IL1, and TNFA, signatures of macrophages, neutrophils, and the complement and coagulation cascades were reduced in Ad26.COV2.S immunized hamsters and macaques compared with sham unvaccinated animals. Similar to our findings, single-cell transcriptomic profiling in hamsters showed proinflammatory pathways, neutrophils and macrophages signatures were enriched in the lungs of sham infected hamsters compared with hamsters that received the mRNA-1273 vaccine [32], suggesting that both mRNA-1273 and Ad26.COV2.S averted proinflammatory pathways induced by SARS-CoV-2 challenge. Similarly, Doremalen and colleagues [33] showed that ChAdOx1 nCoV-19 did not increase serum level of proinflammatory cytokines such as TNF-α, IL-2, IL-4, IL-5 and IL-6 and prevented damage of the lungs in macaques challenged with SARS-CoV-2.

In a Phase 1/2a randomized, clinical study, Ad26.COV2.S vaccination generated robust CD8+, CD4+ T cell, and antibody responses following vaccination [17,27]. Consistent with these clinical data, in the Ad26.COV2.S vaccinated hamsters, we observed upregulation of effector and cytolytic CD8+ and CD4+ T cell signatures. In vaccinated hamsters, we observed upregulation of T helper type 1 (Th1) cell signature and STAT4 targets [29], a transcription factor that has an essential role in the Th1 differentiation and proliferation [29,31,34]. Th1 cells promote cell-mediated immune responses and play an essential role in the host defense against viral infections. In mice, it has been shown that Ad26.COV2.S induced Th1-skewing of T cell response that contribute vaccine protection from severe disease [12]. Similarly, macaques vaccinated with ChAdOx1 nCoV-19 or mRNA-1273 vaccines showed vaccination induced type 1 helper T-cell (Th1)–biased CD4 T-cell responses and low or undetectable Th2 responses [33,35].

We observed upregulation of signatures of T regulatory cell (Tregs) in vaccinated hamsters (**Fig 5A**) suggesting that Tregs might be beneficial in controlling the cytokine storm and the excessive proinflammatory responses induced by SARS-CoV-2. A recent report in COVID-19 patients showed the enrichment of a specific Tregs population in severe COVID-19 patients and suggested that these pathological Tregs may play nefarious roles by suppressing antiviral T cell responses during the severe phase of the disease [30]. Additional work is warranted to explore whether Tregs play a role in Ad26.COV2.S mediated protection and the presence of vaccine induced Tregs.

Consistent with the observation that Ad26.COV2.S protects against SARS-CoV-2, we observed a pronounced increase of signatures of B cell activation, differentiation, and proliferation in vaccinated compared to sham and naïve hamsters at 4 dpi (**Fig 6**). We previously showed that vaccination with Ad26.COV2.S generated high IgG, IgG2a, IgG3, IgM, Fc-receptors FcRγ2, FcRγ3, and FcRγ4 and antibody-dependent complement deposition (ADCD) responses [3]. Our study showed signatures of B cell activation and differentiation induced by Ad26.COV2.S vaccination correlated positively with serum antibody responses. We also showed that serum antibody responses correlated inversely with viral load. Taken together, our data suggest that Ad26.COV2.S vaccination generated potent B cell responses that correlated with serum antibody responses and helped protect hamsters from COVID-19 severe disease.

In summary, this transcriptomic and proteomic study demonstrates that Ad26.COV2.S prevented severe pathological pathways induced by SARS-CoV-2 and suggested potential mechanisms of Ad26 vaccine protection against severe COVID-19 disease. Additional studies are warranted to understand how Ad26.COV2.S stimulates the immune system to elicit protective immune responses.

## Materials and methods

### Ethics Statement

All animal studies were conducted in compliance with all relevant local, state and federal regulations and were approved by the Bioqual Institutional Animal Care and Use Committee.

### Animals and study design

Seventy male and female Syrian golden hamsters (Envigo), 10–12 weeks old, were randomly allocated to groups. All animals were housed at Bioqual. Animals received Ad26 vectors expressing S.dTM.PP or S.PP or sham controls (n  =  10 per group). Animals received a single immunization of 10^10^ or 10^9^ vp Ad26 vectors by the intramuscular route without adjuvant at week 0. At week 4, all animals were challenged with 5.0 × 10^5^ TCID50 or 5.0 × 10^4^ TCID50 SARS-CoV-2, which was derived with one passage from USA-WA1/2020 (NR-52281, BEI Resources). Virus was administered as 100 μl by the intranasal route (50 μl in each nare). Body weights were assessed daily. All immunologic and virologic assays were performed blinded. On day 4, a subset of animals was euthanized for tissue viral loads, pathology and transcriptomics profiling. We performed bulk RNA-Seq on lung frozen tissues from 3 naïve controls, 3 SARS-CoV-2 infected animals, 4 animals vaccinated with Ad26 vectors expressing S.dTM.PP and 5 animals vaccinated with Ad26 vectors expressing S.PP. The immunological, virological and humoral data were previously published by our group [3].

Lung tissue was homogenized in 700 μL of QIAzol (Qiagen) and stored at -80°C until being extracted using the miRNeasy Micro kit (Qiagen) with on-column DNase digestion. RNA quality was assessed using an Agilent Bioanalyzer and ten nanograms of total RNA used as input for library preparation using the SMARTer Stranded Total RNA-Seq V2 Pico Input Mammalian kit (Takara Bio) according to the manufacturer’s instructions. Libraries were validated by capillary electrophoresis on an Agilent 4200 TapeStation, pooled at equimolar concentrations, and sequenced on an Illumina NovaSeq6000 at 100SR, targeting 25–30 million reads per sample. Alignment was performed using STAR version 2.7.3a [36] with the MesAur1.0 (GCF_000349665.1) assembly and annotation of the hamster downloaded from NCBI. Transcript abundance estimates was calculated internal to the STAR aligner using the algorithm of htseq-count as described previously [4]. DESeq2 was used for normalization, producing both a raw and normalized read count table. Differential expression at the gene level were performed by DESeq2 implemented in the DESeq2 R package. A corrected p-value cut-off of 0.05 was used to assess significant genes that were upregulated or down regulated by SARS-Cov2 at day 4 post challenge in sham and vaccinated animals compared to naïve controls and in vaccinated compared to sham animals using Benjamini-Hochberg (BH) method.

### Histopathology and immunohistochemistry

Tissues were fixed in freshly prepared 4% paraformaldehyde for 24 hours, transferred to 70% ethanol, paraffin embedded within 7–10 days, and blocks sectioned at 5 μm. Slides were baked for 30–60 min at 65°C then deparaffinized in xylene and rehydrated through a series of graded ethanol to distilled water. For Iba-1 IHC, heat induced epitope retrieval (HIER) was performed using a pressure cooker on steam setting for 25 minutes in citrate buffer (Thermo; AP-9003-500) followed by treatment with 3% hydrogen peroxide. Slides were then rinsed in distilled water and protein blocked (BioCare, BE965H) for 15 min, followed by rinses in 1x phosphate buffered saline. Primary rabbit anti-Iba-1 antibody (Wako; 019–19741 at 1:4000) was applied for 30 minutes, followed by rabbit Mach-2 HRP-Polymer (BioCare; RHRP520L) for 30 minutes then counterstained with hematoxylin followed by bluing using 0.25% ammonia water. Labeling for Iba-1 was performed on a Biogenex i6000 Autostainer (v3.02). In some cases, Iba-1 staining was performed with Iba-1 at 1:500 (BioCare Cat. No. CP290A; polyclonal), both detected using Rabbit Polink-1 HRP (GBI Labs Cat. No. D13-110). Neutrophil (MPO) and type 1 IFN response (Mx1) was performed with MPO (Dako Cat. No. A0398; polyclonal) at 1:1500 detection using Rabbit Polink-1 HRP, and Mx1 (EMD Millipore Cat. No. MABF938; clone M143/CL143) at 1:1000 detection using Mouse Polink-2 HRP (GBI Labs Cat. No. D37-110). Staining for MPO and Mx1 IHC was performed as previously described using a Biocare intelliPATH autostainer, with all antibodies being incubated for one h at room temperature.

Quantitative image analysis was performed using HALO software (v2.3.2089.27 or v3.0.311.405; Indica Labs) on at least one lung lobe cross-section from each animal. In cases where >1 cross-section was available; each lung lobe was quantified as an individual data point. For Mx1 quantification, the Area Quantification v2.1.3 module was used to determine the percentage of Mx1 protein as a proportion of the total tissue area. For Iba-1 quantification, the Multiplex IHC v2.3.4 module was used to detect Iba-1+ cells and is presented as a proportion of total alveolar tissue. For MPO (neutrophil) quantification, the HALO AI software was first trained to detect the alveolar portion of the lung by excluding blood vessels (>5mm^2^), bronchi, bronchioles, cartilage, and connective tissue; subsequently, the Multiplex IHC v2.3.4 module was used to detect MPO+ cells and is presented as a proportion of total alveolar tissue (PMNs/mm^2^). In all instances, manual curation was performed on each sample to ensure the annotations were accurate and to correct false positives/false negatives.

### ELISA

RBD-specific binding antibodies were assessed by ELISA as described in Tostanoski and colleagues [3]. Briefly, 96-well plates were coated with 1 μg ml−1 of SARS-CoV-2 RBD protein (Aaron Schmidt, Massachusetts Consortium on Pathogen Readiness) or 1 μg ml−1 of SARS-CoV-2 S protein (Sino Biological) in 1Å~Dulbeccoʼs phosphate-buffered saline (DPBS) and incubated at 4°C overnight. After incubation, plates were washed once with wash buffer (0.05% Tween-20 in 1Å~ DPBS) and blocked with 350 μl of casein block per well for 2–3 h at room temperature. After incubation, the block solution was discarded, and plates were blotted dry. Three-fold serial dilutions of heat-inactivated serum in casein block were added to wells, and plates were incubated for one h at room temperature. Plates were washed three times and then subsequently incubated for one h with 0.1 μg ml−1 of anti-hamster IgG HRP (SouthernBiotech) in casein block at room temperature in the dark. Plates were washed three times, and then 100 μl of SeraCare KPL TMB SureBlue Start solution was added to each well; plate development was halted by the addition of 100 μl of SeraCare KPL TMB Stop solution per well. The absorbance at 450 nm was recorded using a VersaMax or Omega microplate reader. ELISA endpoint titers were defined as the highest reciprocal serum dilution that yielded an absorbance two-fold above background.

### SomaScan study design

Twenty-4 outbred Indian-origin adult male and female rhesus macaques (Macaca mulatta) (3–11 years old) were randomly allocated to groups. All macaques were housed at Bioqual. Macaques received a single immunization of 5x10^10^ viral particles of Ad26.COV2.S (n = 12) or sham (n = 12) by the intramuscular route without adjuvant at week 0. At week 6, all macaques were challenged with 5x10^5^ TCID50 SARS-CoV-2 from strains USA-WA1/2020 (BEI Resources; NR-5228) (which was grown in VeroE6 cells and deep sequenced as previously described16) or B.1.351(BEI Resources; NR-54974). The B.1.351 stock was grown in Calu-3 cells and was deep-sequenced, which confirmed the expected sequence identity with no mutations in the S greater than 2.5% frequency and no mutations elsewhere in the virus at greater than 13% frequency. The virus was administered as 1 ml by the intranasal route (0.5 ml in each nare) and 1 ml by the intratracheal route. All immunological, virological, and histopathological studies were performed blinded. Animal studies were conducted in compliance with all relevant local, state, and federal regulations and were approved by the Bioqual Institutional Animal Care and Use Committee.

### SomaScan proteomics profiling

Serum samples were collected at baseline (before immunization) and at days 2 and 10 following challenge from two groups (n = 6 each group) of rhesus macaques that received the Ad26.COV2.S and from another two groups (n = 6 each group) sham unvaccinated animals. All macaques were challenged either with SARS-CoV-2 WA1/2020 or SARS-CoV-2 B.1.351. Serum samples (55ml), five pooled serum controls, and one buffer control were analyzed using the SomaScan Assay Kit for human serum V4.1 (Cat#. 900–00021), measuring expression of 6596 unique human protein targets using 7596 SOMAmer (slow off-rate modified aptamer) reagents, single-stranded DNA aptamers, according to the manufacturer’s standard protocol (SomaLogic; Boulder, CO). The modified aptamer binding reagents1, SomaScan Assay, its performance characteristics, and specificity to human targets have been previously described. The assay used standard controls, including hybridization normalization control sequences used to control for variability in the Agilent microarray readout process as well as five human calibrator control pooled serum replicates and 3 Quality Control (QC) pooled replicates used to mitigate batch effects and verify the quality of the assay run using standard acceptance criteria. The SomaScan Assay is run using 96 well plates; eleven wells are allocated for control samples used to control for batch effects and to estimate the accuracy, precision, and buffer background over time. Five pooled Calibrator replicates, three pooled Quality Control replicates, and three buffer replicates are run on every plate. The readout is performed using Agilent microarray hybridization, scan, and feature extraction technology. Twelve Hybridization Control SOMAmers are added alongside SOMAmers to be measured from the serum samples and controls of each well during the SOMAmer elution step to control for readout variability. The control samples are run repeatedly during assay qualification, and robust point estimates are generated and stored as references for each SOMAmer result for the Calibrator and QC samples. The results are used as references for the SomaScan V4.1 Assay. Plate Calibration is performed by calculating the ratio of the Calibrator Reference relative fluorescence unit (RFU) value to the plate-specific Calibrator replicate median RFU value for each SOMAmer. The resulting ratio distribution is decomposed into a Plate Scale factor defined by the median of the distribution and a vector of SOMAmer-specific Calibration Scale Factors. Normalization of QC replicates and Samples is performed using Adaptive Normalization by Maximum Likelihood (ANML) with point and variance estimates from a normal U.S. population. Post calibration accuracy is estimated using the ratio of the QC reference RFU value to the plate-specific QC replicate median RFU value for each SOMAmer. The resulting QC ratio distribution provides a robust estimate of accuracy for each SOMAmer on every plate. Plate-specific Acceptance Criteria are as follows: Plate Scale Factor between 0.4–2.5 and 85% of QC ratios between 0.8 and 1.2. To analyze the SOMAscan proteomics data, median normalized relative abundances of all measured analytes were imported into R (v3.6.1) using the SomaDataIO package. Principal component analysis (PCA) was performed on log-transformed values using R’s prcomp function, differential protein abundance was evaluated using the limma package (version 3.40.6). RFU were log2 transformed using the Bioconductor package LIMMA. The LIMMA package was used to fit a linear model to each protein and perform (moderated) t-tests. To control the expected proportions of false positives, the FDR for each unadjusted P-value was calculated using the Benjamini and Hochberg method implemented in LIMMA. For data mining and functional analyses, genes that satisfied a p-value <0.05 were selected. Pathway enrichment analysis was performed using GSEA_4.2.2.

### Pathway enrichment analyses

We used the Broad Institute Gene set enrichment analysis software (GSEA) and a compendium of databases of biological and immunological pathways to test the enrichment of pathways and transcription factors (TFs) signatures at 4 dpi in vaccinated, sham, and control hamster groups. Thrombosis signatures were compiled from the MSigDB curated C2 gene sets, IPA ingenuity pathway analysis (https://targetexplorer.ingenuity.com), and were manually curated in-house by checking the individual function of each gene using GeneCard database (https://www.genecards.org/). Cytokines signaling, immune cell signatures and molecular pathways were compiled from the MSigDB Hallmark, C2, C7, and C3 gene sets (https://www.gsea-msigdb.org/gsea/msigdb/collections.jsp), IPA ingenuity pathway analysis https://targetexplorer.ingenuity.com), and the blood transcriptional modules (BTMs)[37]. The GSEA Java desktop program was downloaded from the Broad Institute (http://www.broadinstitute.org/gsea/index.jsp) and used with GSEA Pre-Ranked module parameters (number of permutations: 1,000; enrichment statistic: weighted; 10≤ gene set size ≤5,000). Sample-level enrichment analysis SLEA [38] was used to investigate the enrichment of pathways in each animal. Briefly, the expression of all the genes in a specific pathway was averaged across samples and compared to the average expression of 1,000 randomly generated gene sets of the same size. The resulting z-score was then used to reflect the overall perturbation of each pathway in each sample.

### Canonical pathway and upstream regulator functional analysis

The canonical pathway and upstream regulator functions of the IPA core expression analysis tool (Qiagen) were used to interrogate the lists of genes upregulated or downregulated by SARS-CoV-2 at 4 dpi in sham infected hamsters compared to macaques infected with SARS-CoV-2. Canonical pathways and upstream regulators were considered significant if pathway activation Z-Score ≥ 2 and pathway overlap corrected p-value < 0.05 (using the Benjamini-Hochberg method). Functional analysis of statistically significant gene and protein changes was performed using Ingenuity Pathways Knowledge Base (IPA; Ingenuity Systems). For all gene set enrichment analyses, a right-tailed Fisher’s exact test was used to calculate P-values associated with each biological function and canonical pathway. The calculated z-score signifies whether a gene or protein expression changes for known targets of each regulator are consistent with what is expected from the literature (z > 2, regulator predicted to be activated, z < -2, regulator predicted to inhibited). Additional functional module analyses were performed using Functional module detection (https://hb.flatironinstitute.org/module/). Go term enrichment analysis was performed using the GeneMania database (https://genemania.org/). For GSEA analysis, all significant pathways and molecular signatures, up-or down-regulated in the different groups of hamsters, were selected using a false discovery rate < 20 and a nominal p-value < 0.05. In IPA Global Canonical Pathways (GCP), a multiple-testing corrected p-value was calculated using the Benjamini- Hochberg (BH) method.

### Quantification and statistical analysis

The quality of RNA-Seq raw reads was examined using FastQC (http://www.bioinformatics.babraham.ac.uk/projects/fastqc/) reads were aligned using STAR v2.7.3. Differential expression at the gene level was performed by DESeq2 implemented in the DESeq2 R package. Pathways enrichment and upstream regulators analyses were analyzed through the use of ingenuity pathway analysis (IPA) (QIAGEN Inc., https://www.qiagenbioinformatics.com/products/ingenuitypathway-analysis) and the GSEA Desktop v4.0.3 from (https://www.gsea-msigdb.org/gsea/index.jsp). For data annotation and presentation, we used a collection of tools, including Cytoscape version 3.6.0 (https://cytoscape.org), GeneMANIA version 3.3.1 (http://genemania.org) and Genecards (https://www.genecards.org). Where indicated on the figures, all p-values were adjusted for multiple comparisons using the Benjamini-Hochberg method (BH). Correlations of transcriptomics signatures with viral loads, ELISA binding, and neutralizing titers were measured by correlating the pathway sample level enrichment score (SLEA) with the different outcomes using two-sided Spearman_s rank correlations. The error bands of correlation plots represent 95% confidence limits. All statistical analyses were performed using the R statistical software v3.6.1. S1 Fig was generated using R and BioRender.com. Fig 7 was created with BioRender.com.

## Supporting information

S1 Fig Transcriptomic profiling of naïve, sham unvaccinated, and vaccinated hamsters. **(A)** Study design. 4 groups of Syrian golden hamsters were used in this study from a previously published study by our group [3]. Lung snap-frozen tissues were sampled at day 4 post-challenge for bulk RNA-Seq. **(B)** Expression similarity matrix comparing all animals across the 4 groups. Hierarchical clustering showing uninfected control animals in red in a separate cluster from sham unvaccinated and vaccinated animals. The distance matrix was generated using the R function dist(). In pink color: animals vaccinated with the Ad26.COV2.S; in blue: animals vaccinated with Ad26.S.dTM.PP; in green: sham infected and unvaccinated animals and in red: naïve animals. **(C)** Unsupervised clustering of naïve, infected unvaccinated, and vaccinated groups using PCA plot generated by the prcomp () R function on the two first components where vaccinated groups were shown in blue and pink; naïve uninfected animals in red and sham unvaccinated animals in green. S1 Fig was generated using R and BioRender.com.
(TIF)

S2 Fig SARS-CoV-2 transcripts mRNA levels correlated with viral loads in lung and nares of vaccinated hamsters. A linear regression model of SARS-CoV-2 transcripts (x-axis) normalized reads count as a function of viral load loads (y-axis) in the lung (A) or the nares (B) of vaccinated hamsters. The error bands represent 95% confidence limits. A Spearman correlation and a t-test were performed to assess the significance of the correlations. Each red dot corresponds to an individual animal.
(TIF)

S3 Fig Transcriptomic changes in Ad26 vaccinated hamsters compared to sham unvaccinated and naïve control hamsters. **(A)** Scatter plots of differentially expressed genes in the lung of Ad26.COV2.S, Ad26.SdTM.PP vaccinated and sham-unvaccinated hamsters compared to naive hamsters. Shown are genes that display a significant log2-transformed fold change. Coloration and point size indicate log2-transformed fold changes, red for upregulated genes, and bleu for downregulated genes. The X-axis shows the log 2 transformed fold change expression. The Y-axis shows the -log10 adjusted p-values of genes at 4 dpi. Adjusted P-values (<0.05) were calculated by DEseq2 using Benjamini-Hochberg corrections of Wald test p-values. **(B**) Venn diagram of common and distinct DEGs in Ad26.COV2.S (pink circle) and Ad26.SdTM.PP (blue circle) vaccinated hamsters compared to sham animals at dpi. **(C)** Scatter plot of DEGs upregulated (in red) or downregulated (in blue) in Ad26.COV2.S compared to Ad26.SdTM.PP at 4dpi. The X-axis shows the log 2 transformed fold change expression. The Y-axis shows the -log10 adjusted p-values of genes at 4 dpi. Adjusted P-values (<0.05) were calculated by DEseq2 using Benjamini-Hochberg corrections of Wald test p-values. **(D)** Boxplot representation of the normalized reads counts of the interferon receptors 1 and 2 (Ifnar1 and Ifnar2) and interferon Regulatory Factor 2-Binding Protein 2 in vaccinated, sham-unvaccinated compared to naïve hamsters. Full boxplots correspond to the Ad26.COV2.S high dose and empty boxplots correspond to the Ad26.COV2.S low dose.
(TIF)

S4 Fig Pathological pathways associated with SARS-CoV-2 infection in hamsters and COVID-19 patients were upregulated in hamsters challenged with SARS-CoV-2. **(A-B**) Dot plots of differentially expressed genes increased in infected hamsters at 4 dpi that are upregulated (red) or downregulated (blue) in COVID-19 infected patients or the lung of hamsters infected with SARS-CoV-2. Shown are genes that are increased (red gradient) or decreased (blue gradient) at 4 dpi in sham compared to naïve control animals that were reported in a published SARS-Cov-2 hamsters study [6]. Coloration and point size indicate log2-transformed fold changes and p-values, respectively, of genes at 4 dpi time points relative to control groups (naïve). Adjusted p-values were calculated by DEseq2 using Benjamini-Hochberg corrections of Wald test p-values.
(TIF)

S5 Fig Proinflammatory pathways induced by SARS-CoV-2 in hamsters correlated positively with viral loads at 4 dpi following challenge. Scatter plot of the SLEA score of IL6_JAK_STAT3 and TNF alpha pathways in vaccinated animals at 4 dpi as a function of the ELISA binding titers, neutralizing antibody titers and viral load. The x-axis represents the level of the sample enrichment score of each pathway (SLEA), and the y-axis shows immune responses elicited by Ad26 in vaccinated hamsters. The average expression of the genes within each pathway was calculated using the SLEA z-score method. A linear regression model (blue or red line) fit between SLEA z-score, viral load, and the different antibody responses was performed. A Spearman correlation and a t-test were performed to assess the significance of the correlation between pathways SLEA scores and each response. Each red dot corresponds to an individual animal. Positive correlations were shown in red and negative correlations were shown in blue.
(TIF)

S6 Fig Correlation of macrophages and neutrophils signatures with binding and neutralizing titers and viral loads in vaccinated hamsters. Scatter plot of the SLEA score of macrophages pathways in vaccinated animals at 4 dpi as a function of the ELISA binding titers, neutralizing antibody titers, and viral load. The x-axis represents the level of the sample enrichment score of each pathway (SLEA), and the y-axis shows immune responses elicited by Ad26 in vaccinated hamsters. P-value and the Spearman correlation coefficient were shown for each plot. Each red dot corresponds to an individual animal. Positive correlations were shown in red and negative correlations were shown in blue.
(TIF)

S7 Fig Correlation of the complement cascade and thrombosis-associated signatures with binding and neutralizing titers and viral loads in vaccinated hamsters. Scatter plot of the SLEA score of thrombosis-associated pathways in vaccinated animals at 4 dpi as a function of the ELISA binding titers, neutralizing antibody titers, and viral load. The x-axis represents the level of the sample enrichment score of each pathway (SLEA), and the y-axis shows immune responses elicited by Ad26 in vaccinated hamsters. P-value and the Spearman correlation coefficient were shown for each plot. Each red dot corresponds to an individual animal. Positive correlations were shown in red and negative correlations were shown in blue.
(TIF)

S8 Fig T regulatory markers expressed in patients with severe COVID-19 disease increased in sham unvaccinated hamsters. Heatmap of Tregs markers upregulated (red) or downregulated (blue) in sham unvaccinated hamsters compared with Ad26.COV2.S vaccinated hamsters or control naïve animals. Color gradient represents the log 2 transformation of gene fold change expression. Only significantly increased or decreased genes were shown (BH-corrected p value threshold of 0.05).
(TIF)

S9 Fig Early transcriptomic signatures of CD8 (A) and TH1 (B) T cells correlated with binding and neutralizing titers following Ad26.CoV-2.S immunization in hamsters. (TIF)

S10 Fig Early proteomics signatures of BCR signaling and antigen presentation correlated with binding (ELISA) and neutralizing (pVNA) titers following Ad26.CoV-2.S immunization in monkeys. (TIF)

S1 Table SARS-CoV-2 transcripts summarized in Fig 1A. (XLSX)

S2 Table Expression of genes on day 4 following SARS-CoV-2 challenge in hamsters summarized in S4 Fig. (XLSX)

## References

[1] Chandrashekar A. et al., "SARS-CoV-2 infection protects against rechallenge in rhesus macaques," *Science*, vol. 369, no. 6505, pp. 812–817, August 14 2020 2020, doi: 10.1126/science.abc4776 32434946 PMC7243369

[2] Munster V. J. et al., "Respiratory disease in rhesus macaques inoculated with SARS-CoV-2," *Nature*, vol. 585, no. 7824, pp. 268–272, Sep 2020, doi: 10.1038/s41586-020-2324-7 32396922 PMC7486227

[3] Tostanoski L. H. et al., "Ad26 vaccine protects against SARS-CoV-2 severe clinical disease in hamsters," *Nat Med*, vol. 26, no. 11, pp. 1694–1700, Nov 2020, doi: 10.1038/s41591-020-1070-6 32884153 PMC7671939

[4] Aid M. et al., "Vascular Disease and Thrombosis in SARS-CoV-2-Infected Rhesus Macaques," *Cell*, vol. 183, no. 5, pp. 1354–1366 e13, Nov 25 2020, doi: 10.1016/j.cell.2020.10.005 33065030 PMC7546181

[5] Munoz-Fontela C. et al., "Animal models for COVID-19," *Nature*, vol. 586, no. 7830, pp. 509–515, Oct 2020, doi: 10.1038/s41586-020-2787-6 32967005 PMC8136862

[6] Nouailles G. et al., "Longitudinal omics in Syrian hamsters integrated with human data unravel complexity of moderate immune responses to SARS-CoV-2," *bioRxiv*, 2020, doi: 10.1101/2020.12.18.423524

[7] Oa Donnell K. L. et al., "Pathogenic and transcriptomic differences of emerging SARS-CoV-2 variants in the Syrian golden hamster model," *bioRxiv*, Jul 12 2021, doi: 10.1101/2021.07.11.451964 34758415 PMC8572342

[8] Sia S. F. et al., "Pathogenesis and transmission of SARS-CoV-2 in golden hamsters," *Nature*, vol. 583, no. 7818, pp. 834–838, Jul 2020, doi: 10.1038/s41586-020-2342-5 32408338 PMC7394720

[9] Imai M. et al., "Syrian hamsters as a small animal model for SARS-CoV-2 infection and countermeasure development," *Proc Natl Acad Sci U S A*, vol. 117, no. 28, pp. 16587–16595, Jul 14 2020, doi: 10.1073/pnas.2009799117 32571934 PMC7368255

[10] Merad M. and Martin J. C., "Pathological inflammation in patients with COVID-19: a key role for monocytes and macrophages," *Nat Rev Immunol*, vol. 20, no. 6, pp. 355–362, Jun 2020, doi: 10.1038/s41577-020-0331-4 32376901 PMC7201395

[11] Abbink P. et al., "Comparative seroprevalence and immunogenicity of six rare serotype recombinant adenovirus vaccine vectors from subgroups B and D," *J Virol*, vol. 81, no. 9, pp. 4654–63, May 2007, doi: 10.1128/JVI.02696-06 17329340 PMC1900173

[12] Bos R. et al., "Ad26 vector-based COVID-19 vaccine encoding a prefusion-stabilized SARS-CoV-2 Spike immunogen induces potent humoral and cellular immune responses," *NPJ Vaccines*, vol. 5, p. 91, 2020, doi: 10.1038/s41541-020-00243-x 33083026 PMC7522255

[13] "<Cryo-EM structure of the 2019-nCoV spike in the prefusion conformation.pdf>." 10.1126/science.abb2507 PMC7164637 32075877

[14] Mercado N. B. et al., "Single-shot Ad26 vaccine protects against SARS-CoV-2 in rhesus macaques," *Nature*, vol. 586, no. 7830, pp. 583–588, Oct 2020, doi: 10.1038/s41586-020-2607-z 32731257 PMC7581548

[15] van der Lubbe J. E. M. et al., "Ad26.COV2.S protects Syrian hamsters against G614 spike variant SARS-CoV-2 and does not enhance respiratory disease," *NPJ Vaccines*, vol. 6, no. 1, p. 39, Mar 19 2021, doi: 10.1038/s41541-021-00301-y 33741993 PMC7979827

[16] Yu J. et al., "Protective efficacy of Ad26.COV2.S against SARS-CoV-2 B.1.351 in macaques," *Nature*, Jun 23 2021, doi: 10.1038/s41586-021-03732-8 34161961 PMC8373608

[17] Sadoff J. et al., "Safety and Efficacy of Single-Dose Ad26.COV2.S Vaccine against Covid-19," *N Engl J Med*, vol. 384, no. 23, pp. 2187–2201, Jun 10 2021, doi: 10.1056/NEJMoa2101544 33882225 PMC8220996

[18] Acharya D., Liu G., and Gack M. U., "Dysregulation of type I interferon responses in COVID-19," *Nat Rev Immunol*, vol. 20, no. 7, pp. 397–398, Jul 2020, doi: 10.1038/s41577-020-0346-x 32457522 PMC7249038

[19] Blanco-Melo D. et al., "Imbalanced Host Response to SARS-CoV-2 Drives Development of COVID-19," *Cell*, vol. 181, no. 5, pp. 1036–1045 e9, May 28 2020, doi: 10.1016/j.cell.2020.04.026 32416070 PMC7227586

[20] Mahmudpour M., Roozbeh J., Keshavarz M., Farrokhi S., and Nabipour I., "COVID-19 cytokine storm: The anger of inflammation," *Cytokine*, vol. 133, p. 155151, Sep 2020, doi: 10.1016/j.cyto.2020.155151 32544563 PMC7260598

[21] Mazzoni A. et al., "Impaired immune cell cytotoxicity in severe COVID-19 is IL-6 dependent," *J Clin Invest*, vol. 130, no. 9, pp. 4694–4703, Sep 1 2020, doi: 10.1172/JCI138554 32463803 PMC7456250

[22] Liao M. et al., "Single-cell landscape of bronchoalveolar immune cells in patients with COVID-19," *Nat Med*, vol. 26, no. 6, pp. 842–844, Jun 2020, doi: 10.1038/s41591-020-0901-9 32398875

[23] Hoang T. N. et al., "Baricitinib treatment resolves lower airway inflammation and neutrophil recruitment in SARS-CoV-2-infected rhesus macaques," *bioRxiv*, Sep 16 2020, doi: 10.1101/2020.09.16.300277 33278358 PMC7654323

[24] Ackermann M. et al., "Pulmonary Vascular Endothelialitis, Thrombosis, and Angiogenesis in Covid-19," *N Engl J Med*, vol. 383, no. 2, pp. 120–128, Jul 9 2020, doi: 10.1056/NEJMoa2015432 32437596 PMC7412750

[25] Williamson E. J. et al., "Factors associated with COVID-19-related death using OpenSAFELY," *Nature*, vol. 584, no. 7821, pp. 430–436, Aug 2020, doi: 10.1038/s41586-020-2521-4 32640463 PMC7611074

[26] Roh J. et al., "Plasma Proteomics of COVID-19 Associated Cardiovascular Complications: Implications for Pathophysiology and Therapeutics," *Res Sq*, Jun 8 2021, doi: 10.21203/rs.3.rs-539712/v1 35530264 PMC9067411

[27] Sadoff J. et al., "Interim Results of a Phase 1-2a Trial of Ad26.COV2.S Covid-19 Vaccine," *N Engl J Med*, Jan 13 2021, doi: 10.1056/NEJMoa2034201 33440088 PMC7821985

[28] McMahan K. et al., "Correlates of protection against SARS-CoV-2 in rhesus macaques," *Nature*, Dec 4 2020, doi: 10.1038/s41586-020-03041-6 33276369 PMC7906955

[29] Nishikomori R., Usui T., Wu C. Y., Morinobu A., O’Shea J. J., and Strober W., "Activated STAT4 has an essential role in Th1 differentiation and proliferation that is independent of its role in the maintenance of IL-12R beta 2 chain expression and signaling," *J Immunol*, vol. 169, no. 8, pp. 4388–98, Oct 15 2002, doi: 10.4049/jimmunol.169.8.4388 12370372

[30] Galvan-Pena S. et al., "Profound Treg perturbations correlate with COVID-19 severity," *bioRxiv*, Dec 15 2020, doi: 10.1101/2020.12.11.416180 34433692 PMC8449354

[31] Thieu V. T. et al., "Signal transducer and activator of transcription 4 is required for the transcription factor T-bet to promote T helper 1 cell-fate determination," *Immunity*, vol. 29, no. 5, pp. 679–90, Nov 14 2008, doi: 10.1016/j.immuni.2008.08.017 18993086 PMC2768040

[32] Meyer M. et al., "mRNA-1273 efficacy in a severe COVID-19 model: attenuated activation of pulmonary immune cells after challenge," *bioRxiv*, Jan 25 2021, doi: 10.1101/2021.01.25.428136 33532780 PMC7852274

[33] van Doremalen N. et al., "ChAdOx1 nCoV-19 vaccine prevents SARS-CoV-2 pneumonia in rhesus macaques," *Nature*, vol. 586, no. 7830, pp. 578–582, Oct 2020, doi: 10.1038/s41586-020-2608-y 32731258 PMC8436420

[34] Svensson A., Tunback P., Nordstrom I., Shestakov A., Padyukov L., and Eriksson K., "STAT4 regulates antiviral gamma interferon responses and recurrent disease during herpes simplex virus 2 infection," *J Virol*, vol. 86, no. 17, pp. 9409–15, Sep 2012, doi: 10.1128/JVI.00947-12 22718836 PMC3416106

[35] Corbett K. S. et al., "Evaluation of the mRNA-1273 Vaccine against SARS-CoV-2 in Nonhuman Primates," *N Engl J Med*, vol. 383, no. 16, pp. 1544–1555, Oct 15 2020, doi: 10.1056/NEJMoa2024671 32722908 PMC7449230

[36] Dobin A. et al., "STAR: ultrafast universal RNA-seq aligner," *Bioinformatics*, vol. 29, no. 1, pp. 15–21, Jan 1 2013, doi: 10.1093/bioinformatics/bts635 23104886 PMC3530905

[37] Li S. et al., "Molecular signatures of antibody responses derived from a systems biology study of five human vaccines," *Nat Immunol*, vol. 15, no. 2, pp. 195–204, Feb 2014, doi: 10.1038/ni.2789 24336226 PMC3946932

[38] Gundem G. and Lopez-Bigas N., "Sample-level enrichment analysis unravels shared stress phenotypes among multiple cancer types," *Genome Medicine*, vol. 4(3):28, 2012/03/29 2012, doi: 10.1186/gm327 22458606 PMC3446278

