# Supplementary figures and images for: Ad26.COV2.S prevents upregulation of SARS-CoV-2 induced pathways of inflammation and thrombosis in hamsters and rhesus macaques
Source: PLoS Pathog. 2022 Apr 8;18(4):e1009990. doi: 10.1371/journal.ppat.1009990 (PMC9020736; doi:10.1371/journal.ppat.1009990)

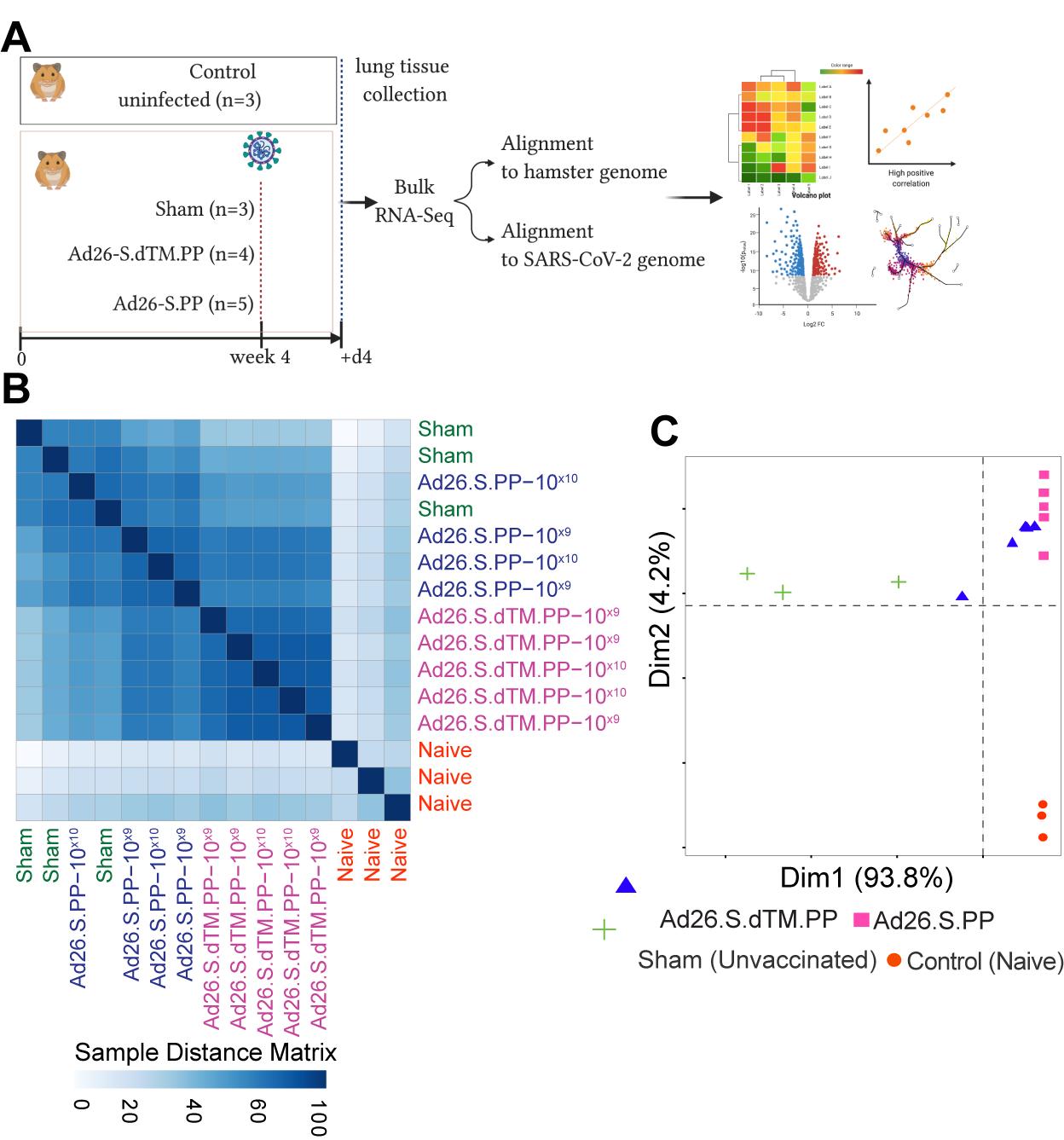

Supplement: S1 Fig — (A) Study design. 4 groups of Syrian golden hamsters were used in this study from a previously published study by our group [3]. Lung snap-frozen tissues were sampled at day 4 post-challenge for bulk RNA-Seq. (B) Expression similarity matrix comparing all animals across the 4 groups. Hierarchical clustering showing uninfected control animals in red in a separate cluster from sham unvaccinated and vaccinated animals. The distance matrix was generated using the R function dist(). In pink color: animals vaccinated with the Ad26.COV2.S; in blue: animals vaccinated with Ad26.S.dTM.PP; in green: sham infected and unvaccinated animals and in red: naïve animals. (C) Unsupervised clustering of naïve, infected unvaccinated, and vaccinated groups using PCA plot generated by the prcomp () R function on the two first components where vaccinated groups were shown in blue and pink; naïve uninfected animals in red and sham unvaccinated animals in green. S1 Fig was generated using R and BioRender.com. (TIF) [file ppat.1009990.s001.tif]

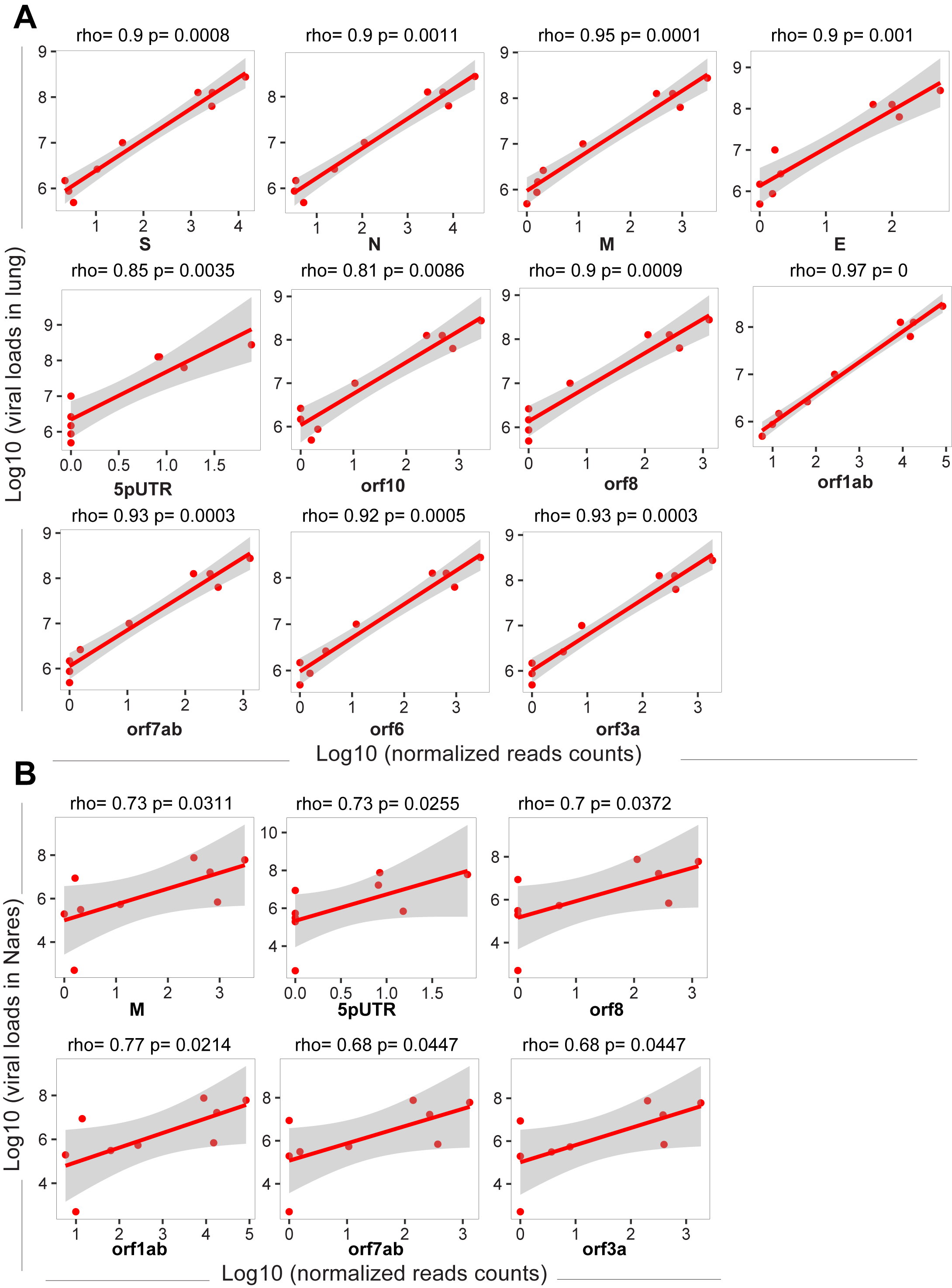

Supplement: S2 Fig — A linear regression model of SARS-CoV-2 transcripts (x-axis) normalized reads count as a function of viral load loads (y-axis) in the lung (A) or the nares (B) of vaccinated hamsters. The error bands represent 95% confidence limits. A Spearman correlation and a t-test were performed to assess the significance of the correlations. Each red dot corresponds to an individual animal. (TIF) [file ppat.1009990.s002.tif]

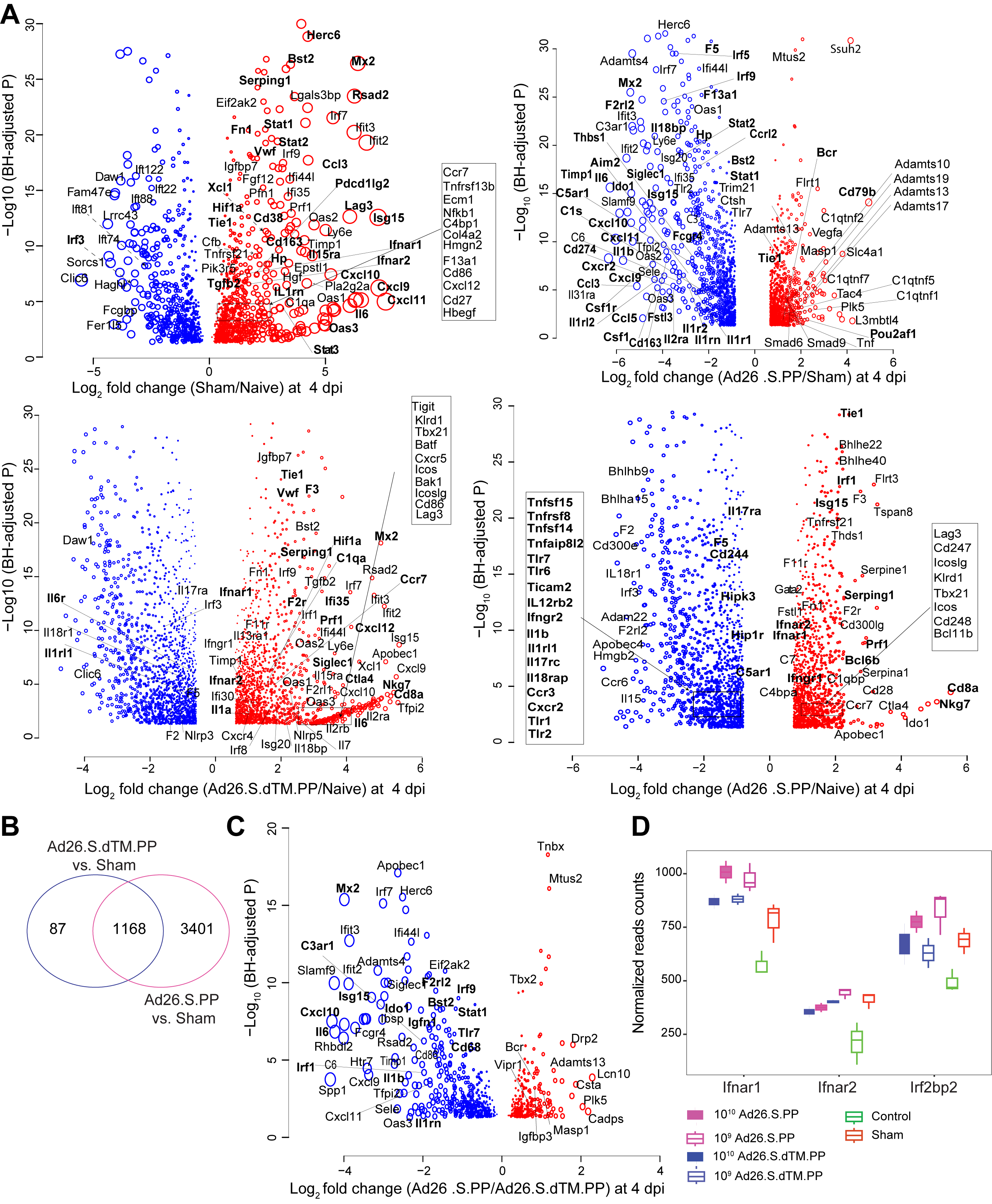

Supplement: S3 Fig — (A) Scatter plots of differentially expressed genes in the lung of Ad26.COV2.S, Ad26.SdTM.PP vaccinated and sham-unvaccinated hamsters compared to naive hamsters. Shown are genes that display a significant log2-transformed fold change. Coloration and point size indicate log2-transformed fold changes, red for upregulated genes, and bleu for downregulated genes. The X-axis shows the log 2 transformed fold change expression. The Y-axis shows the -log10 adjusted p-values of genes at 4 dpi. Adjusted P-values (<0.05) were calculated by DEseq2 using Benjamini-Hochberg corrections of Wald test p-values. (B) Venn diagram of common and distinct DEGs in Ad26.COV2.S (pink circle) and Ad26.SdTM.PP (blue circle) vaccinated hamsters compared to sham animals at dpi. (C) Scatter plot of DEGs upregulated (in red) or downregulated (in blue) in Ad26.COV2.S compared to Ad26.SdTM.PP at 4dpi. The X-axis shows the log 2 transformed fold change expression. The Y-axis shows the -log10 adjusted p-values of genes at 4 dpi. Adjusted P-values (<0.05) were calculated by DEseq2 using Benjamini-Hochberg corrections of Wald test p-values. (D) Boxplot representation of the normalized reads counts of the interferon receptors 1 and 2 (Ifnar1 and Ifnar2) and interferon Regulatory Factor 2-Binding Protein 2 in vaccinated, sham-unvaccinated compared to naïve hamsters. Full boxplots correspond to the Ad26.COV2.S high dose and empty boxplots correspond to the Ad26.COV2.S low dose. (TIF) [file ppat.1009990.s003.tif]

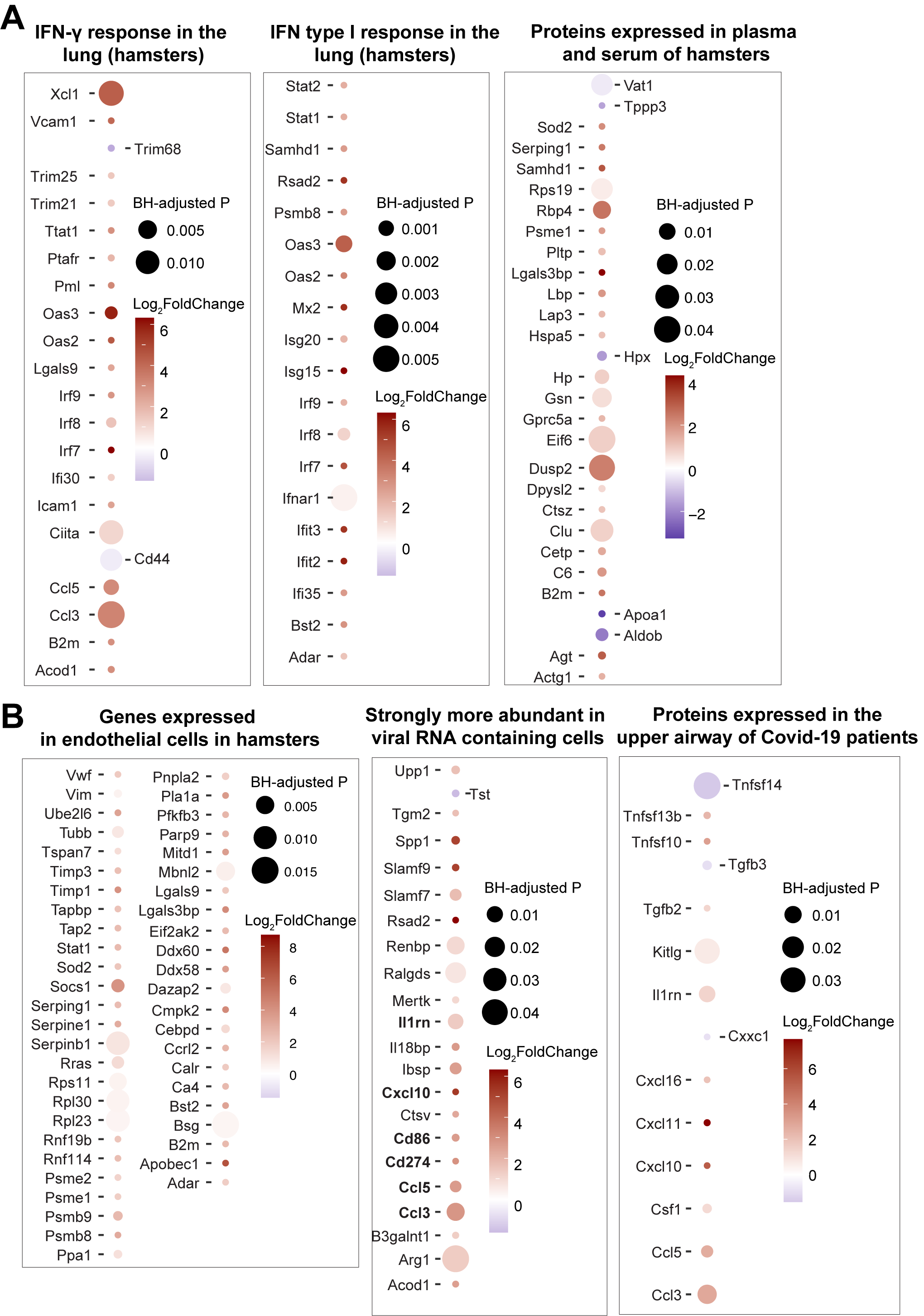

Supplement: S4 Fig — (A-B) Dot plots of differentially expressed genes increased in infected hamsters at 4 dpi that are upregulated (red) or downregulated (blue) in COVID-19 infected patients or the lung of hamsters infected with SARS-CoV-2. Shown are genes that are increased (red gradient) or decreased (blue gradient) at 4 dpi in sham compared to naïve control animals that were reported in a published SARS-Cov-2 hamsters study [6]. Coloration and point size indicate log2-transformed fold changes and p-values, respectively, of genes at 4 dpi time points relative to control groups (naïve). Adjusted p-values were calculated by DEseq2 using Benjamini-Hochberg corrections of Wald test p-values. (TIF) [file ppat.1009990.s004.tif]

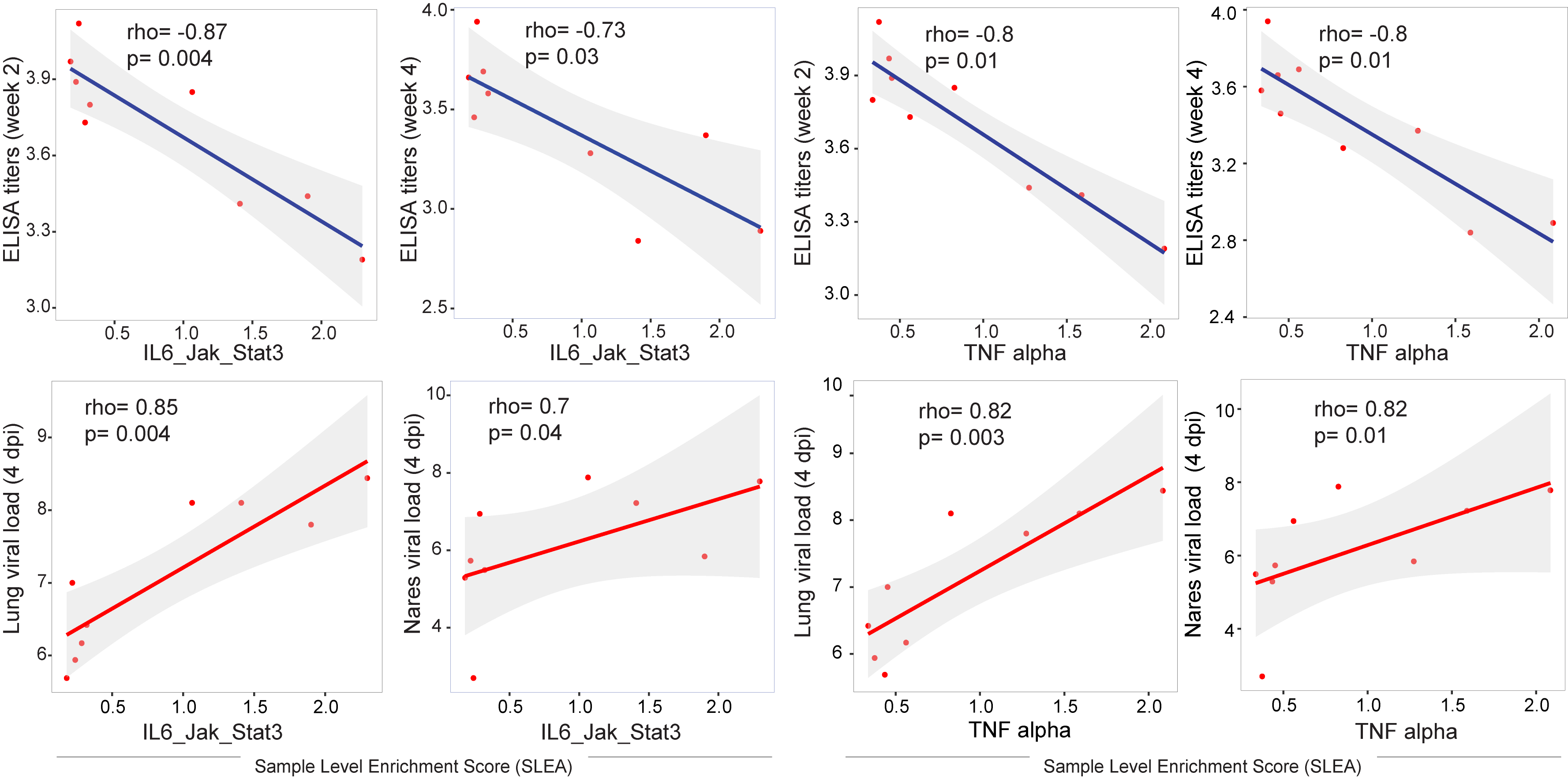

Supplement: S5 Fig — Scatter plot of the SLEA score of IL6_JAK_STAT3 and TNF alpha pathways in vaccinated animals at 4 dpi as a function of the ELISA binding titers, neutralizing antibody titers and viral load. The x-axis represents the level of the sample enrichment score of each pathway (SLEA), and the y-axis shows immune responses elicited by Ad26 in vaccinated hamsters. The average expression of the genes within each pathway was calculated using the SLEA z-score method. A linear regression model (blue or red line) fit between SLEA z-score, viral load, and the different antibody responses was performed. A Spearman correlation and a t-test were performed to assess the significance of the correlation between pathways SLEA scores and each response. Each red dot corresponds to an individual animal. Positive correlations were shown in red and negative correlations were shown in blue. (TIF) [file ppat.1009990.s005.tif]

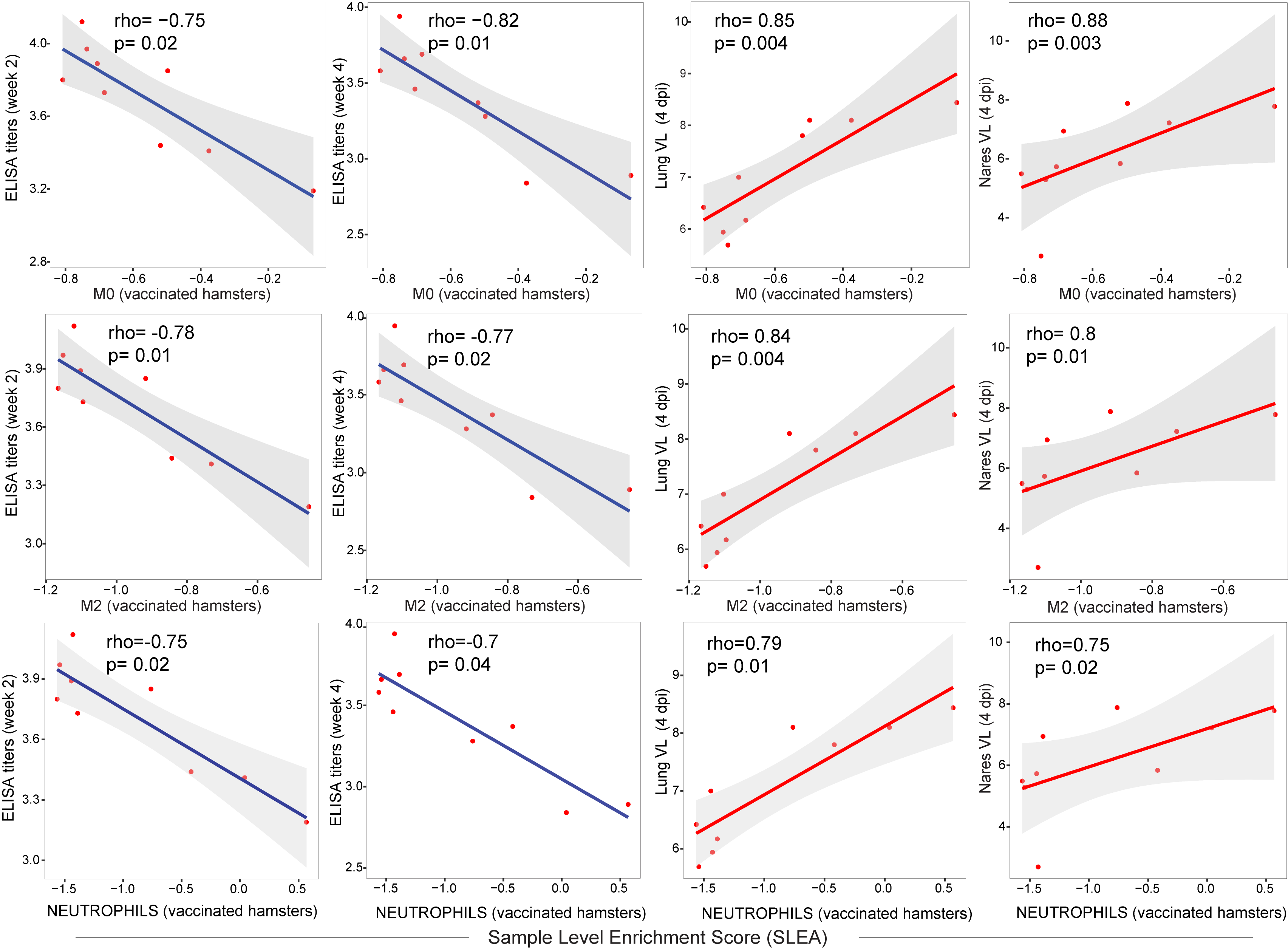

Supplement: S6 Fig — Scatter plot of the SLEA score of macrophages pathways in vaccinated animals at 4 dpi as a function of the ELISA binding titers, neutralizing antibody titers, and viral load. The x-axis represents the level of the sample enrichment score of each pathway (SLEA), and the y-axis shows immune responses elicited by Ad26 in vaccinated hamsters. P-value and the Spearman correlation coefficient were shown for each plot. Each red dot corresponds to an individual animal. Positive correlations were shown in red and negative correlations were shown in blue. (TIF) [file ppat.1009990.s006.tif]

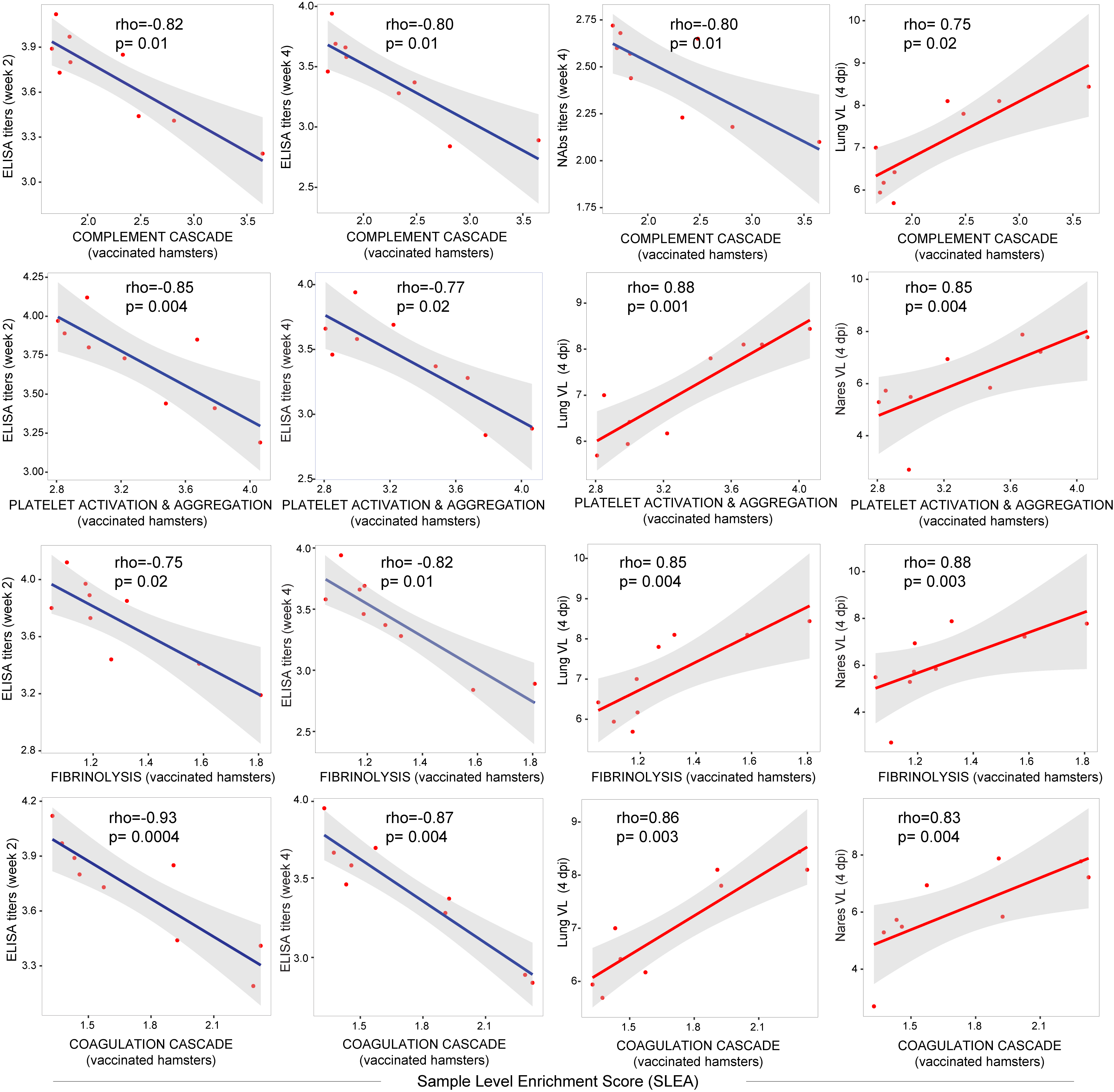

Supplement: S7 Fig — Scatter plot of the SLEA score of thrombosis-associated pathways in vaccinated animals at 4 dpi as a function of the ELISA binding titers, neutralizing antibody titers, and viral load. The x-axis represents the level of the sample enrichment score of each pathway (SLEA), and the y-axis shows immune responses elicited by Ad26 in vaccinated hamsters. P-value and the Spearman correlation coefficient were shown for each plot. Each red dot corresponds to an individual animal. Positive correlations were shown in red and negative correlations were shown in blue. (TIF) [file ppat.1009990.s007.tif]

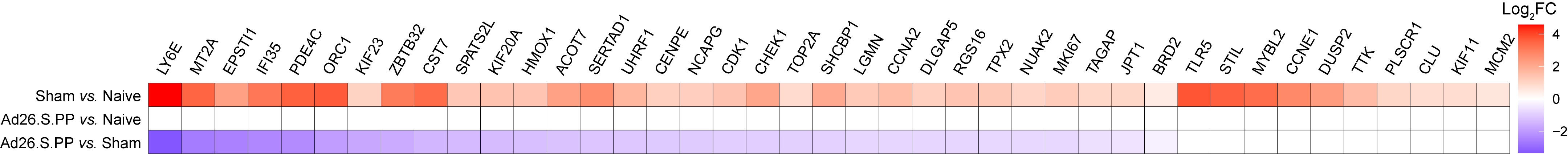

Supplement: S8 Fig — Heatmap of Tregs markers upregulated (red) or downregulated (blue) in sham unvaccinated hamsters compared with Ad26.COV2.S vaccinated hamsters or control naïve animals. Color gradient represents the log 2 transformation of gene fold change expression. Only significantly increased or decreased genes were shown (BH-corrected p value threshold of 0.05). (TIF) [file ppat.1009990.s008.tif]

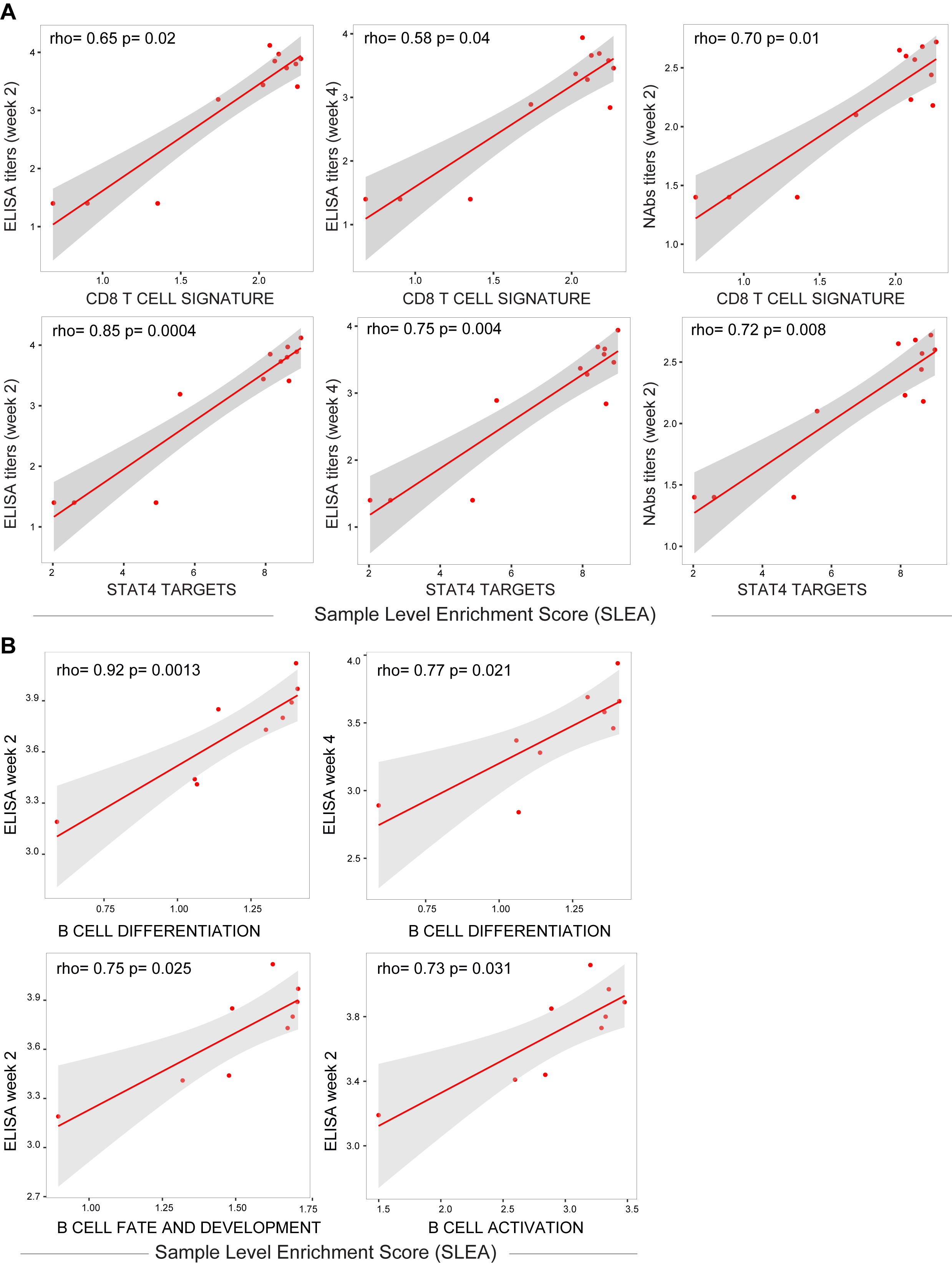

Supplement: S9 Fig — (TIF) [file ppat.1009990.s009.tif]

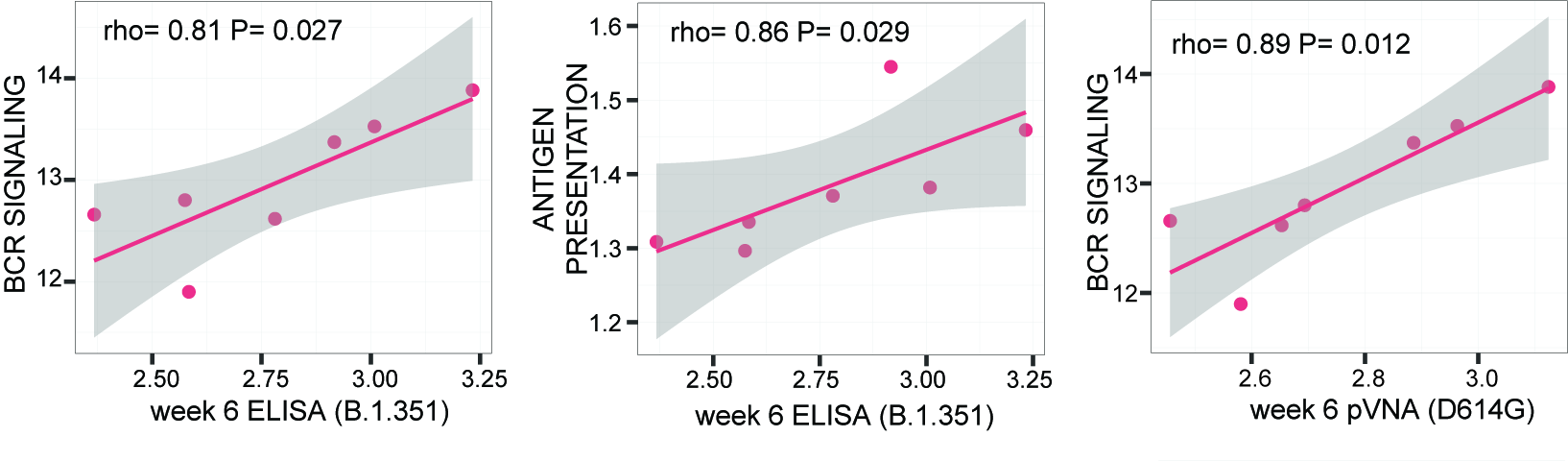

Supplement: S10 Fig — (TIF) [file ppat.1009990.s010.tif]
